# Angiosperms Are Unique among Land Plant Lineages in the Occurrence of Key Genes in the RNA-Directed DNA Methylation (RdDM) Pathway

**DOI:** 10.1093/gbe/evv171

**Published:** 2015-09-02

**Authors:** Lu Ma, Andrea Hatlen, Laura J. Kelly, Hannes Becher, Wencai Wang, Ales Kovarik, Ilia J. Leitch, Andrew R. Leitch

**Affiliations:** ^1^School of Biological and Chemical Sciences, Queen Mary University of London, United Kingdom; ^2^Department of Molecular Epigenetics, Institute of Biophysics, Academy of Sciences of the Czech Republic, Brno, Czech Republic; ^3^Department of Comparative Plant and Fungal Biology Royal Botanic Gardens, Kew, Richmond, Surrey, United Kingdom

**Keywords:** chromatin modification, DNA methylation, evolution, RNA-directed DNA methylation, seed plants

## Abstract

The RNA-directed DNA methylation (RdDM) pathway can be divided into three phases: 1) small interfering RNA biogenesis, 2) de novo methylation, and 3) chromatin modification. To determine the degree of conservation of this pathway we searched for key genes among land plants. We used OrthoMCL and the OrthoMCL Viridiplantae database to analyze proteomes of species in bryophytes, lycophytes, monilophytes, gymnosperms, and angiosperms. We also analyzed small RNA size categories and, in two gymnosperms, cytosine methylation in ribosomal DNA. Six proteins were restricted to angiosperms, these being NRPD4/NRPE4, RDM1, DMS3 (defective in meristem silencing 3), SHH1 (SAWADEE homeodomain homolog 1), KTF1, and SUVR2, although we failed to find the latter three proteins in *Fritillaria persica*, a species with a giant genome. Small RNAs of 24 nt in length were abundant only in angiosperms. Phylogenetic analyses of Dicer-like (DCL) proteins showed that DCL2 was restricted to seed plants, although it was absent in *Gnetum gnemon* and *Welwitschia mirabilis*. The data suggest that phases (1) and (2) of the RdDM pathway, described for model angiosperms, evolved with angiosperms. The absence of some features of RdDM in *F. persica* may be associated with its large genome. Phase (3) is probably the most conserved part of the pathway across land plants. DCL2, involved in virus defense and interaction with the canonical RdDM pathway to facilitate methylation of CHH, is absent outside seed plants. Its absence in *G. gnemon*, and *W. mirabilis* coupled with distinctive patterns of CHH methylation, suggest a secondary loss of DCL2 following the divergence of Gnetales.

## Introduction

The first land plants appeared in the fossil record around 470–480 Ma (Kenrick and Crane 1997; Wellman et al. 2003) and the species which survive today can be broadly divided into four major groups: 1) the nonvascular plants, which comprise bryophytes (liverworts, mosses, and hornworts); 2) the lycophytes, the earliest diverging extant group of vascular plants; 3) the monilophytes, which include the horsetails (*Equisetum*), whisk ferns (e.g., *Psilotum*), ophioglossoid ferns (e.g., *Ophioglossum*), and true ferns; and 4) the seed plants comprising angiosperms (flowering plants) and gymnosperms (naked-seed plants). With the huge increase in genomic data available for species belonging to these different groups, it has become clear that the genome dynamics of each group are distinctive (reviewed in Leitch and Leitch [2012, 2013]). Here, we explore the composition of the epigenetic machinery in representatives of these major groups and suggest how the differences encountered might have played a role in shaping their genome dynamics. In particular, we compare and contrast the genes involved in controlling the RNA-directed DNA methylation (RdDM) pathway, with a particular emphasis on angiosperms and gymnosperms, but including representatives of the other land plant groups to determine directionality of change in the evolution of this pathway.

Gymnosperms comprise approximately 780 species and are represented by four distinct lineages, the cycads (Cycadales), *Ginkgo* (Ginkgoales), Gnetales, and Coniferales (conifers). Our understanding of their genome structure, and the epigenetic processes that regulate their genomes, is largely restricted to Pinaceae (Leitch and Leitch 2012). Outside this family understanding is minimal, and in most cases missing entirely. Nevertheless, despite this dearth of knowledge, we do know that gymnosperms have reduced frequencies of polyploidy in all but *Ephedra* (Gnetales; Leitch and Leitch 2012) and there is some evidence of alternative mechanisms to regulate the evolution of their genome, for example, different epigenetic marks associated with heterochromatin (Fuchs et al. 2008), higher levels of transcription of retrotransposons in conifers than angiosperms (Morse et al. 2009; Parchman et al. 2010), and lower levels of unequal recombination to remove the long-terminal repeats (LTRs) from LTR retrotransposons (Nystedt et al. 2013). Such differences have been postulated to have fundamentally shaped patterns of genome evolution in seed plants (Leitch and Leitch 2012).

It is widely recognized that the diversity of genome sizes in land plants arises from differences in the accumulation of repetitive elements, including tandem and dispersed repeats, as well as the frequency of polyploidy, or whole-genome duplication, in the lineages’ ancestry. This article focuses on the evolution of mechanisms that control the accumulation of repeats and searches for differences in these mechanisms between representative species of the major land plant lineages.

**Fig. 1.—:**
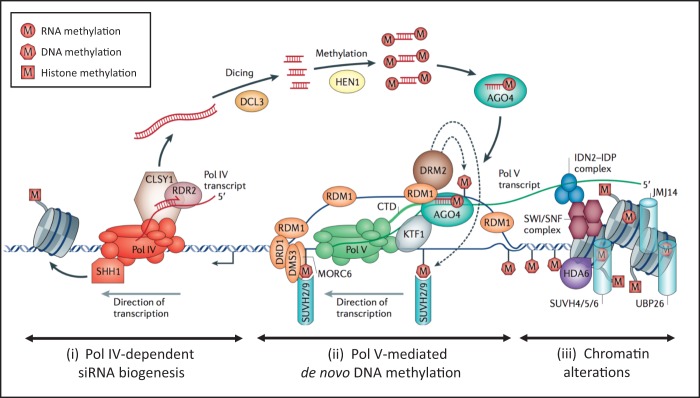
The RdDM pathway, taken from Matzke and Mosher (2014). The genes involved are shown, and details are given, in the source reference. The pathway is divided into three key phases, 1) Pol IV-dependent siRNA biogenesis, 2) Pol V-mediated de novo DNA methylation, and 3) chromatin alterations. An overview of their activity is given in the introduction and the full names of genes given in supplementary table S1, Supplementary Material online.

Regulation of repeats in angiosperms broadly falls into two categories: 1) RdDM de novo methylation and 2) maintenance methylation pathways, the latter involving genes which play a role in CG and CHG methylation. This work focuses on the RdDM pathway, leading to the heterochromatinization of repeats in angiosperms, as summarized in figure 1, which outlines the canonical pathway. Briefly, the RdDM pathway can be divided into three phases: 1) RNA polymerase IV (Pol IV)-dependent small interfering RNA (siRNA) biogenesis, 2) RNA polymerase V (Pol V)-mediated de novo DNA methylation, and 3) chromatin alteration or modification (review in Matzke and Mosher [2014]). In the first of these, Pol IV activity synthesizes RNA transcripts, which are made double stranded by RNA-dependent RNA polymerase 2 (RDR2) and “diced” or cut into 24 nt siRNAs using Dicer-like 3 (DCL3) endonuclease. These siRNAs are then complexed with the argonaute (AGO) protein AGO4 and directed back to the nucleus. Then the siRNAs, through sequence homology, are targeted back to DNA repeats. In phase (2) Pol V is involved in the further transcription of repeats in association with the diced 24 nt siRNA to facilitate RdDM in a little understood process. Finally, in phase (3), genes involved in histone modification and chromatin folding heterochromatize the DNA sequence. This process then “seeds” methylation, which may spread into surrounding genic regions and become extended and supplemented by the activities of the maintenance methylation pathways which typically involve the recognition and full methylation of hemimethylated CG and CHG sites by methyltransferase 1 (MET1) and chromomethylase 3 (CMT3) DNA methyltransferases, respectively, and of CHH by CMT2 (Matzke and Mosher 2014).

**Fig. 2.—:**
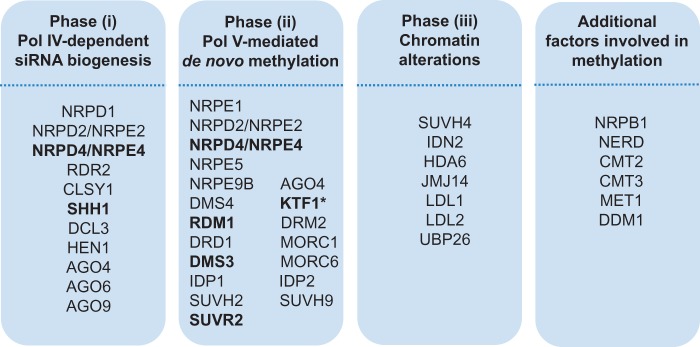
Key genes of the RdDM pathway taken from Matzke and Mosher (2014) and Matzke et al. (2015) that have been analyzed. Genes given in bold were not detected by us outside angiosperms. The genes are grouped into three categories in line with the three phases of chromatin remodeling shown in figure 1.

The vast majority of research into the genes involved in the RdDM pathway in plants has been conducted in *Arabidopsis thaliana*. Thus to search for the occurrence of these genes across the different land plant groups, we retrieved the key genes and their paralogues from *Ar. thaliana* (fig. 2) and used these to search for their occurrence in cluster groups of OrthoMCL from publically available proteome sequence databases of bryophytes, lycophytes, monilophytes, representatives from the gymnosperm lineages, the early diverging angiosperm *Amborella trichopoda*, and some model angiosperms (e.g., *Zea mays*). In addition, because gymnosperms have significantly larger genomes than most angiosperms, we hypothesized that this may be due to different activities of RdDM. To test that hypothesis we also searched the transcriptome of *Fritillaria persica*, an angiosperm with a particularly large genome (1C = 41.21 pg; Kelly et al. 2015).

## Materials and Methods

### Data Used to Search for Orthologues

A flow diagram illustrating our bioinformatic approaches is shown in supplementary figure S1, Supplementary Material online. Twelve proteomes from representative taxa of land plants were selected comprising 1) the angiosperms *Ar. thaliana* L. (Heynh.), *Am. trichopoda*, Baill. *F. persica* L., *Z. mays* L.; 2) the gymnosperms *Ginkgo biloba* L., *Gnetum gnemon* L., *Picea abies* (L.) H.Karst., *Pinus taeda* L., *Welwitschia mirabilis* Hook.f.; 3) the monilophyte *Pteridium aquilinum* L.Kuhn.; 4) the lycophyte *Selaginella moellendorffii* Hieron; and 5) the bryophyte *Physcomitrella patens* (Hedw.) Bruch and Schimp.

Proteome data for eight of these species were retrieved from public databases (see table 1). For the remaining four species we derived proteome data from transcriptomes. For *Pt. aquilinum* we used the transcriptome data from Li et al. (2014). For *Gn. gnemon*, we downloaded Illumina raw reads from NCBI SRA archive (ERR364403) (table 1). For *F. persica* and *W. mirabilis*, we used new transcriptomic data (see below), and reads from the mRNA library of *F. persica*, *Gn. gnemon*, and *W. mirabilis* were de novo assembled (table 1) using Trinity (version r2013-02-25; Grabherr et al. 2011) with default settings. TransDecoder was then used to identify the protein-coding regions from the de novo assembled contigs using default settings and keeping sequences longer than 100 amino acids.

**Table 1:** Transcriptomes, Proteomes, and sRNA Data Used in This Study

Species	Abbreviation	Source of Proteomes	No. of Proteins	Source of sRNA	Tissue for sRNA	No. of sRNAs (18–26 nt)
**Angiosperms**						
*Amborella trichopoda*	ATRI	Phytozome v10 (http://phytozome.jgi.doe.gov/)	26,846	http://smallrna.udel.edu/data.php	Leaves	4,003,853
*Arabidopsis thaliana*	ATHA	Phytozome v10 (http://phytozome.jgi.doe.gov/)	35,386	GEO (GSM154370)	Leaves	15,831
*Fritillaria persica*	FPER	Trinity_assembled (see link^a^)	62,452	—	—	—
*Zea mays*	ZMAY	Phytozome v10 (http://phytozome.jgi.doe.gov/)	88,760	http://smallrna.udel.edu/data.php	Leaves	3,662,565
**Gymnosperms**						
*Ginkgo biloba*	GBIL	ftp://ftp.plantbiology.msu.edu/pub/data/MPGR/Ginkgo_biloba/	65,468	http://smallrna.udel.edu/data.php	Leaves	3,623,537
*Gnetum gnemon*	GMON	Trinity-assembled (see link^a^) ERR364403	26,782	—	—	—
*Welwitschia mirabilis*	WMIR	Trinity-assembled (see link^a^)	18,255	See link^a^	Leaves	56,649,017
*Picea abies*	PABI	http://congenie.org	66,632	http://smallrna.udel.edu/data.php	Needles	3,010,087
*Pinus taeda*	PTAE	http://pinegenome.org/pinerefseq/ (v1.01)	64,809	—	—	—
**Monilophytes**						
*Pteridium aquilinum*	PAQU	NCBI Transcriptome Shotgun Assembly (GASP00000000.1)	23,332	—	—	—
**Lycophytes**						
*Selaginella moellendorffii*	SMOE	Phytozome v10 (http://phytozome.jgi.doe.gov/)	22,285	GEO (GSM176654)	Above-ground tissues	1,30,240
**Bryophytes**						
*Physcomitrella patens*	PPAT	Phytozome v10 (http://phytozome.jgi.doe.gov/)	42,392	GEO (GSM115095)	Proto-nemata	97,999

Note—All URLs were last accessed on September 10, 2015.

^a^https://goo.gl/PrNKfB.

### RNA Sequencing

For *F. persica* we obtained transcriptomic data by extracting mRNA from leaves as in Becher et al. (2014). The transcriptome of *F. persica* was sequenced by the Centre of Genomic Research at the University of Liverpool, United Kingdom using HiSeq2000 (100 bp paired-end reads). Total RNA of *W. mirabilis* from fresh leaf fragments was extracted using a mirVana miRNA isolation kit (Life Technology) following the manufacturer’s instructions. Both transcriptome and small RNA (sRNA) sequencing of *W. mirabilis* was conducted by BGI, Shenzhen, China on the HiSeq2000 platform (library fragment size for *W. mirabilis* transcriptome sequencing was 270 bp with 91 bp paired-end reads; library fragment size for *W. mirabilis* sRNA sequencing was 107 bp with 50 bp single-end reads).

### Finding OrthoMCL Gene Groups of the RdDM Pathway in Land Plants

Proteomes from the 12 representative land plant taxa were filtered using the pipeline OrthoMCL (v2.0.9; Li et al. 2003; Fischer et al. 2011) and the number of “good” protein sequences, as defined by OrthoMCL (using default settings), for each species is shown in table 1. We searched the proteomes against each other using BLASTp, and then with OrthoMCL. We generated OrthoMCL groups of proteins (clusters) based on similarity, keeping matches with *E* values <1e^−^^5^ and ≥50% match along the protein length. The MCL algorithm was used to generate the OrthoMCL clusters of proteins with an inflation value of 1.5. To find orthologues of genes in the RdDM pathway, we first retrieved the protein sequences listed in figure 2 (see also supplementary table S1, Supplementary Material online, for the full names of each protein) from *Ar. thaliana*, and used these to extract orthologous and paralagous proteins from the OrthoMCL clusters of the other 11 species. The protein information retrieved for DMS3 (defective in meristem silencing 3), KTF1, DCL, and RDM1 (RNA-directed DNA methylation 1) is given in supplementary tables S2–S5, Supplementary Material online, respectively, and all protein sequences from each group are given in FASTA format in the Supplementary data file S1, Supplementary Material online. Custom Python scripts (available on request) were used to extract the protein groups and corresponding protein sequences for each locus in the RdDM pathway based on the gene names used for *Ar. thaliana* (reference proteins, supplementary table S1, Supplementary Material online).

For six of the RdDM genes, the OrthoMCL groups did not contain sequences from any of the nonangiosperm species analyzed. For these genes, we also searched for orthologues in the OrthoMCL Viridiplantae database (http://www.orthomcl.org/orthomcl/, last accessed September 10, 2015) by BLASTp. The OrthoMCL Viridiplantae database includes data from the following eight plant species—angiosperms: *Ar. thaliana*, *Oryza sativa*, and *Ricinus communis*; bryophytes: *Ph. Patens*; and green algae: *Chlamydomonas reinhardtii*, *Micromonas* sp. RCC299, *Ostreococcus tauri*, and *Volvox carteri*.

### Generation of DCL Protein Trees

Using the approach above, DCL putative orthologues from the 12 representative land plant species were extracted from the OrthoMCL output by searching for OrthoMCL groups containing each of the four *Arabidopsis* DCL genes (i.e., DCL1, accession AT1G01040; DCL2, accession AT3G03300; DCL3, accession AT3G43920; DCL4, accession AT5G20320). Protein domains of all sequences were analyzed by scanning predicted protein sequences against the Pfam protein database (http://pfam.xfam.org/search, last accessed September 10, 2015). When more than one splice variant was present for a gene, only the longest protein sequence was kept for analysis. When more than one incomplete protein from the same species had the same domains, we kept the longest variant. Protein sequences that passed these selection criteria were aligned using MUSCLE with default parameters (version 3.8.31; Edgar 2004) and trimmed using trimAl (version 1.2rev59; Capella-Gutiérrez et al. 2009) with the setting “automated1” to remove regions with an excessive amount of missing data or poorly aligned regions. We used ProtTest (version 3.4; Darriba et al. 2011) to select the best model (LG+I+G) based on Bayesian Information Criterion, and RAxML (version 7.4.2; Stamatakis 2006) to build the phylogenetic trees, performing 1,000 bootstrap replicates and using the following options: -p 12345, -f a, -c 4, -x 12345.

### Generation of RDM1 Protein Tree

Phylogenetic analysis of protein sequences from the RDM1 locus was performed in the same way as described above for DCL. However, only four RDM1 protein sequences were isolated from the OrthoMCL results, all from angiosperms. Consequently, we also searched for putative homologues by performing BLASTp searches against the NCBI Protein Reference Sequence database (http://www.ncbi.nlm.nih.gov/protein, last accessed September 10, 2015), retaining all protein matches with an *E* value <1e^−^^5^ and ≥50% identity.

### sRNA Analysis

Most of the sRNA data analyzed were downloaded from public databases (table 1) and comprised reads that had already been trimmed to remove adapter sequences. For *W. mirabilis*, sRNAs were sequenced here (see above). Custom Python scripts were used to obtain the length of each sRNA sequence within the 18–26 nt size range.

### Southern Hybridization

Purified genomic DNAs of *Gi. biloba*, *Gn. gnemon* and, as a control, the angiosperm *Nicotiana tabacum* L. (∼2 μg/sample) were digested with the restriction enzymes MspI, HpaII, BstNI or ScrFI and separated by gel electrophoresis on a 0.9% (w/v) agarose gel. The gels were then alkali-blotted onto Hybond-XL membranes (GE Healthcare, Little Chalfont, United Kingdom) and hybridized with a ^32^P-labeled DNA probe (DekaLabel kit, MBI, Fermentas, Vilnius, Lithuania) for the 18 S ribosomal RNA (rRNA) gene according to protocols described in Kovarik et al. (2005). After washing (2 × 5 min with 2 × SSC, 0.1% SDS at room temperature followed by 2 × 15 min with 0.6 × SSC, 0.1% SDS, 65 °C), the hybridization bands were visualized with a PhosphorImager (Typhoon 9410, GE Healthcare, PA) and the data quantified by ImageQuant software (GE Healthcare, PA). The 18S probe was a 300-bp fragment (fig. 6*a*) obtained by amplification of the 18S rRNA gene of the gymnosperm *Cycas revoluta* Thunb. using primers described further below.

### Bisulphite Sequencing

Modification of DNA with bisulphite was carried out with an EpiTect kit (Qiagen, Germany) using 1.3 μg of genomic DNA from leaves. The primers used amplified the coding strand of the 18S rRNA gene subregion shown in figure 6*a* and did not discriminate between methylated and nonmethylated templates. The primer sequences were as follows: 18SBIS forward: 5′-TATGAGTYTGGTAATTGGAATG-3′; 18SBIS reverse: 5′-TTTAARCACTCTAATTTCTTCAAA-3′. The polymerase chain reaction (total volume 25 μl) used 1.0 μl of bisulphite-converted DNA as the template, 4 nmol of each dNTP, 8 pmol of each primer, and 0.8 U of Kapa *Taq* DNA polymerase (Kapabiosystems). Cycling conditions were as follows: initial denaturation (94 °C/3 min); 35 cycles of (94 °C/20 s; 55 °C/20 s; 72 °C/20 s); and a final extension (72 °C/10 min). The resulting c. 300 bp products were separated by gel electrophoresis, purified and cloned into a TA vector (pDrive, Qiagen). In total, 22 and 18 clones were sequenced from *Gn. gnemon* and *Gi. biloba*, respectively. After trimming of primers the 241 bp-long sequences were aligned and statistically evaluated using CyMATE software (Hetzl et al. 2007).

## Results

### OrthoMCL Clustering

The proteomes of 12 species were compiled to include representative taxa from all four major land plant lineages. Together these 12 taxa generated between 18,255 (*W. mirabilis*) and 88,760 (*Z. mays*) protein sequences, summing to a total of 543,399 proteins that were clustered into 55,357 OrthoMCL groups (containing both paralogues and orthologues) using OrthoMCL (table 1).

We found OrthoMCL groups for all 31 genes/gene families listed in figure 2 which represent genes belonging to the three phases of the canonical RdDM pathway, namely: 1) Pol IV-dependent siRNA biogenesis, 2) Pol V-mediated de novo DNA methylation, and 3) chromatin alterations (fig. 1) together with additional factors also involved in cytosine methylation. OrthoMCL groups of nine proteins or families involved in the RdDM pathway, namely the NRPD2/NRPE2, NRPE9B, NRPB1, RDR, DCL, HEN, AGO, HDA, and UBP contained sequences from all 12 of the species analyzed, indicating high levels of conservation for these loci across land plants (highlighted in green in supplementary table S1, Supplementary Material online). MET1, which codes for Methyltransferase 1, and DDM1 (Decreased DNA methylation 1), which is a chromatin remodeler protein, were also found in all analyzed species.

Putative homologues of DMS3 were found in all plants except *Pin. taeda* and *Ph. patens* (supplementary table S2, Supplementary Material online). However, closer analysis revealed that the protein was either unusually long, indicative of SMC proteins involved in chromatin remodeling (Matzke et al. 2015), or so short that it was not possible to distinguish DMS3 from SMC homologues. Consequently, proteins greater than 700 amino acids and less than 150 amino acids were removed. Proteins with a histidine kinase-like ATPase motif, present in the SMC-related protein AtGMI1 (Böhmdorfer et al. 2011) but not in DMS3, were also removed. This left only putative DMS3 OrthoMCL groups in seed plants. These proteins were aligned using T-Coffee (Notredame et al. 2000) and the alignment quality was assessed using Transitive Consistence Score (TCS, Chang et al. 2015). Four proteins had poor alignment (TCS ≤ 16, see supplementary table S2, Supplementary Material online) and these, together with one isoform of the protein from *Z*, *mays*, were removed from the analysis, leaving seven sequences, all from seed plants. The SMC-related protein from *Ar. thaliana* (GMI1_AT5G24280) was added to the alignment. Phylogenetic analysis of these eight sequences revealed two groups, one containing angiosperms, the other gymnosperms, each being separated by GMI1 from *Ar. thaliana* (supplementary fig. S2, Supplementary Material online). Thus, sequences from gymnosperms cannot be distinguished from SMC-related proteins, and only in angiosperms can we confidently identify DMS3-like sequences, consistent with Matzke et al. (2015).

We searched the data for OrthoMCL groups that were found only in angiosperms and so missing in all other land plant groups and found, in addition to DMS3, a further five proteins in this category: NRPD4/NRPE4, SHH1 (SAWADEE homeodomain homolog 1), RDM1, SUVR2, and KTF1 (all shown in bold in figure 2 and highlighted in blue in supplementary table S1, Supplementary Material online). All six proteins belong to phase (1) and/or (2) of the RdDM pathway. From the 12 proteomes included in the OrthoMCL analysis, only the eudicot *Ar. thaliana* yielded sequences for SUVR2 and SHH1 (but see below).

We extended our proteome analysis to include the OrthoMCL Viridiplantae database, which contains data from six additional plant species not analyzed above (*O. sativa*, *R. communis*, *Ch. reinhardtii*, *Macromonas* sp., *O. tauri*, and *V. carteri*). We focused our search on identifying homologues of the six proteins found only in angiosperms (see above). Using this extended approach SUVR2 and SHH1 were found in all three angiosperm species listed in the OrthoMCL Viridiplantae database, including the monocot *O. sativa*, showing that these gene families are not restricted to eudicots. In *Ar. thaliana* we identified three putative homologues of the SHH1-family and five of SUVR-family, whereas in both *O. sativa* and *R. communis* we identified one putative homologue in each.

Beyond the angiosperms, no sequences with homology to NRPD4/NRPE4, SHH1, RDM1, and SUVR2 were found in gymnosperms, monilophytes or lycophytes, but putative KTF1 (KOW domain-containing transcription factor 1; a synonym of SPT5L) homologues were found in the bryophyte *Ph. patens*, and the green algae *Micromonas* sp. RCC299 and *V. carteri* in the OrthoMCL Viridiplantae database. Because OrthoMCL relies on low thresholds of BLAST similarity (*E* values <1 e^−^^5^ and ≥50% match along the protein length), we further characterized these proteins, by searching for NGN and KOW domains, together with the WG/GW motifs characteristic of KTF1 (He, Hsu, Zhu, et al. 2009; Matzke et al. 2015) (supplementary fig. S3, Supplementary Material online). We failed to find NGN and KOW domains outside the angiosperms (supplementary table S3, Supplementary Material online). We also noticed that while the putative KTF1 sequences in the angiosperm *F. perisca* contained both NGN and KOW domains (Matzke et al. 2015), they lacked GW/WG motifs, perhaps because the protein is a partial assembly (supplementary fig. S3 and table S3, Supplementary Material online).

In summary, the combined results from our analysis indicated that NRPD4/NRPE4, SHH1, RDM1, KTF1, DMS3, and SUVR2 are restricted to angiosperms.

### Phylogenetic Relationships between Members of the DCL Family Proteins

In *Ar. thaliana* it is known that there are four paralogues in the DCL family, these are DCL1 which generates 21 nt microRNAs (miRNAs), DCL2 generating 22 nt siRNAs from viral sequences, DCL3 involved in RdDM and generating 24 nt siRNAs (fig. 1) and DCL4, generating 21 nt siRNAs and trans-acting siRNAs. In *Ar. thaliana*, expression levels of each DCL family member are similar and at medium levels in most tissues (Zimmermann et al. 2004), so we might expect to detect the presence of orthologues in other species, if they are present.

**Fig. 3.—:**
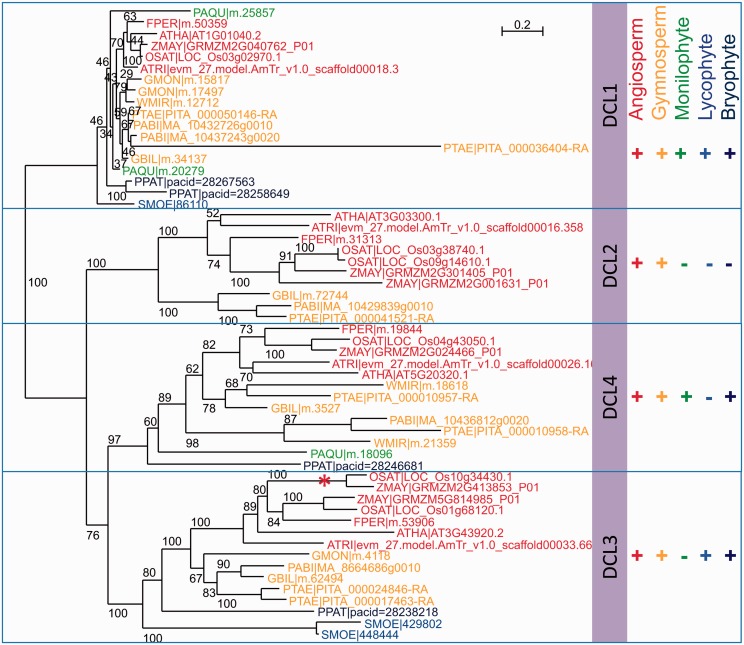
Phylogenetic relationships between DCL sequences showing four distinct DCL clades (DCL1-4). The DCL3b (or DCL5, Song et al. 2012; Fei et al. 2013) clade is labeled with a red asterisk. The (+/−) symbols indicate the land plant group in which each DCL paralogue was found. *Physcomitrella patens* (PPAT), *Selaginella moellendorffii* (SMOE), *Pteridium aquilinum* (PAQU), *Pinus taeda* (PTAE), *Picea abies* (PABI), *Welwitschia mirabilis* (WMIR), *Gnetum gnemon* (GMON), *Ginkgo biloba* (GBIL), *Amborella trichopoda* (ATRI), *Fritillaria persica* (FPER), *Zea mays* (ZMAY), *Oryza sativa* (OSAT), and *Arabidopsis thaliana* (ATHA).

From the 12 species that are the focus of this study, a total of 84 proteins formed a “DCL family group.” They included the four DCL family members in *Ar. thaliana* (DCL1–DCL4), which, when complete, should each exceed 1,300 amino acids. Protein domains of all sequences were analyzed by scanning against the Pfam protein database (http://pfam.xfam.org/search, last accessed September 10, 2015). When more than one splice variant, or size variant was present, only the longest protein sequence was kept for analysis. All sequence variants were kept, leaving 56 protein sequences (supplementary table S4, Supplementary Material online). Phylogenetic relationships between the DCL family members are shown in figure 3. The sequences group into four strongly supported clades, and in each clade there is an *Ar. thaliana* DCL member, as expected. This enabled us to label the clades DCL1–DCL4. Recently, it has been noted that there are two distinct clades of DCL3-like sequences in monocots, called DCL3a and DCL3b, the later renamed DCL5 (Margis et al. 2006; Song et al. 2012; Fei et al. 2013), and represented by two DCL3 clades each containing *Z. mays* and *O. sativa* sequences (see fig. 3). Table 2 summarizes the number of sequences (paralogues) for each of the four DCL family members across the 12 species analyzed. All species had proteins related to DCL1. Of particular note was DCL2, which was absent outside the seed plants (i.e., angiosperms and gymnosperms) and, perhaps significantly, also absent in the two gymnosperms analyzed belonging to Gnetales (*W. mirabilis* and *Gn. gnemon*). There were also isolated absences of DCL3 (in *W. mirabilis* and *Pt. aquilinum*) and DCL4 (in *Gn. gnemon* and *S. moellendorffii*). It may also be significant that we found only two DCL4 domains in *F. persica* (Helicase C and Dicer-dimer domains, out of the nine DCL domains considered, supplementary table S4, Supplementary Material online).

**Table 2:** Numbers of Paralogues in Each of the DCL Family Members

Species	Total Number	DCL1	DCL2	DCL3	DCL4
**Angiosperms**					
*Arabidopsis thaliana*	4	1	1	1	1
*Zea mays*	6	1	2	2 (DCL3/5)	1
*Fritillaria persica*	4	1	1	1	1
*Amborella trichopoda*	4	1	1	1	1
**Gymnosperms**					
*Ginkgo biloba*	4	1	1	1	1
*Picea abies*	5	2^a^	1	1	1
*Pinus taeda*	7	2	1	2	2^a^
*Gnetum gnemon*	3	2^a^	0	1	0
*Welwitschia mirabilis*	3	1	0	0	2
**Monilophytes**					
*Pteridium aquilinum*	3	2^a^	0	0	1
**Lycophytes**					
*Selaginella moellendorffii*	3	1	0	2	0
**Bryophytes**					
*Physcomitrella patens*	4	2	0	1	1

^a^The assembled proteins are incomplete, and based on their sequences and domains present (see supplementary table S4, Supplementary Material online) they may represent a single protein.

### RDM1 Family

**Fig. 4.—:**
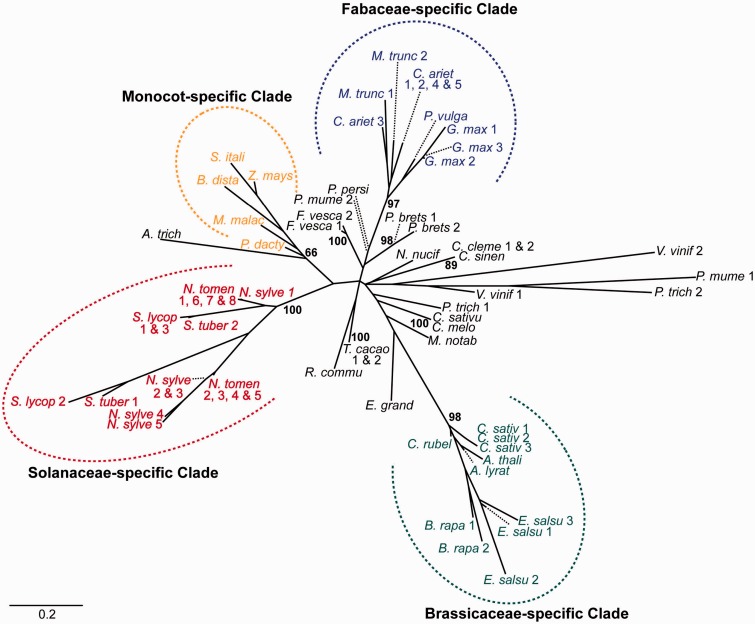
Unrooted phylogenetic tree depicting relationships between RDM1-like protein sequences from angiosperms. All protein sequences used to build the tree were extracted from the NCBI Protein Reference Sequence database by BLASTp (see supplementary table S5, Supplementary Material online, for Genbank accession numbers used). *Amborella trichopoda* (*A. trich*), *Arabidopsis lyrata* subsp*. lyrata* (*A. lyrat*), *Arabidopsis thaliana* (*A. thali*), *Brachypodium distachyon* (*B. dista*), *Brassica rapa* (*B. rapa*), *Camelina sativa* (*C. sativ*), *Capsella rubella* (*C. rubel*), *Cicer arietinum* (*C. ariet*), *Citrus clementine* (*C. cleme*), *Citrus sinensis* (*C. sinen*), *Cucumis melo* (*C. melo*), *Cucumis sativus* (*C. sativu*), *Eucalyptus grandis* (*E. grand*), *Eutrema salsugineum* (*E. salsu*), *Fragaria vesca* subsp. *vesca* (*F. vesca*), *Glycine max* (*G. max*), *Medicago truncatula* (*M. trunc*), *Morus notabilis* (*M. notab*), *Musa acuminata* subsp. *malaccensis* (*M. malac*), *Nelumbo nucifera* (*N. nucif*), *Nicotiana sylvestris* (*N. sylve*), *Nicotiana tomentosiformis* (*N. tomen*), *Phaseolus vulgaris* (*P. vulga*), *Phoenix dactylifera* (*P. dacty*), *Populus trichocarpa* (*P. trich*), *Prunus mume* (*P. mume*), *Prunus persica* (*P. persi*), *Pyrus* x *bretschneideri* (*P. brets*), *Ricinus communis* (*R. commu*), *Setaria italica* (*S. itali*), *Solanum lycopersicum* (*S. lycop*), *Solanum tuberosum* (*S. tuber*), *Theobroma cacao* (*T. cacao*), *Vitis vinifera* (*V. vinif*), *Zea mays* (*Z. mays*). Where there were multiple sequences from a single species, a number follows the taxon abbreviation. Numbers on branches show bootstrap support values for key nodes discussed in the text; due to reasons of space, the support values for other nodes have been omitted.

OrthoMCL clustering revealed one RDM1 orthologue each for *Ar. thaliana*, *F. persica*, *Z. mays*, and *Am. trichopoda*. To better understand the evolution of RDM1, we BLAST-searched the NCBI Protein Reference Sequence database to look for further sequences with similarity to RDM1 and found 68, all from 35 angiosperm species (comprising one early-diverging, 3 monocot, and 12 eudicot families; supplementary table S5, Supplementary Material online). These were aligned and used to build a phylogenetic tree of sequence relationships (fig. 4). Within the RDM1 phylogenetic tree, three family specific clades were recovered; one comprising all the sequences from Brassicaceae species, another containing all the sequences from Fabaceae species, and a third made up of sequences from species belong to Solanaceae. These three eudicot clades were very strongly supported (bootstrap support >95%). For five genera from four further eudicot families with two or more sequences (i.e., *Citrus* [Rutaceae], *Cucumis* [Cucurbitaceae], *Theobroma* [Malvaceae], and *Fragaria* and *Pyrus* [both Rosaceae]), the sequences clustered by genus with strong support. A further clade was identified which contained all RDM1 sequences from monocot species, but it lacked strong support.

### Length Distribution of sRNA

**Fig. 5.—:**
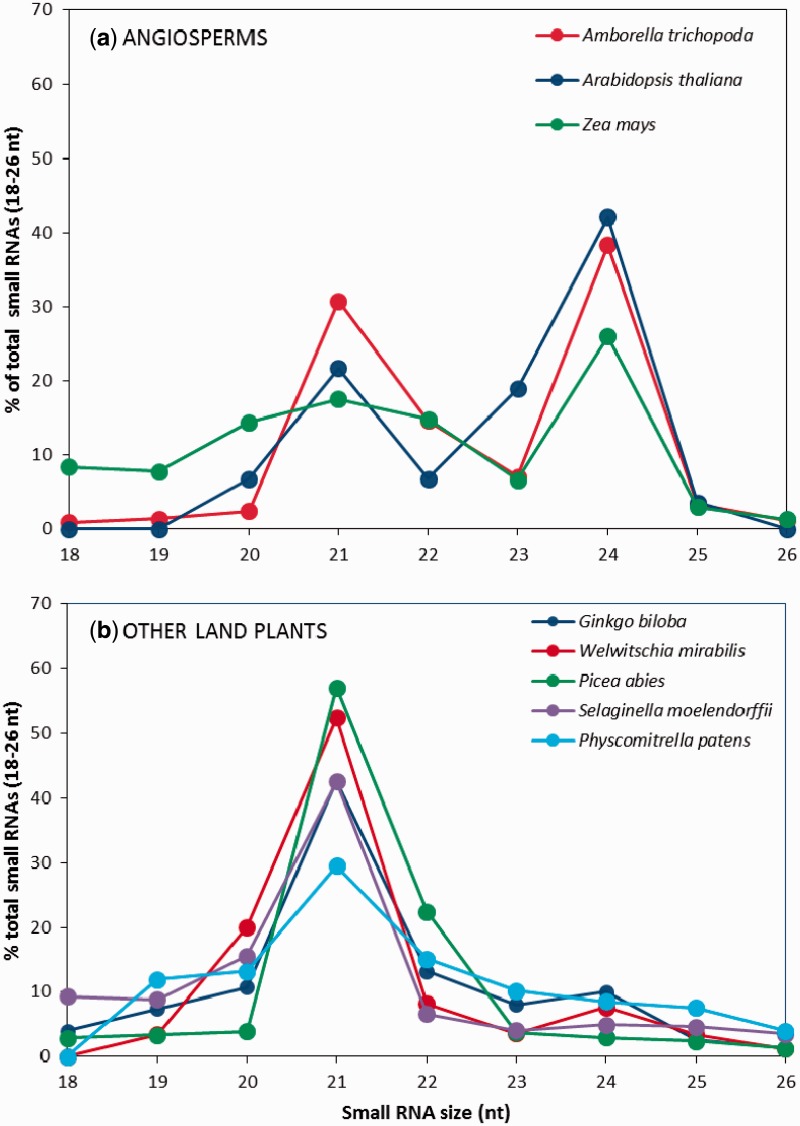
Length distribution of sRNA sequences from (*a*) three angiosperm species and (*b*) five other land plant species listed in table 1. The percentage of total reads for each size class is plotted.

Because the total number of available sequences differed in the eight sRNA data sets examined (from angiosperms: *Am. trichopoda*, *Z. mays*, *Ar. thaliana*; from gymnosperms: *Gi. biloba*, *Pic. abies*, *W. mirabilis*; from lycophytes: *S. moellendorffii*; and from bryophytes *Ph. patens*; see table 1), we plotted the percentage of sRNA sequences belonging to each size category (fig. 5). The most abundant category was 24 nt for all angiosperms (fig. 5*a*). In contrast, for all other land plants analyzed the 21 nt sRNA size category was most abundant (fig. 5*b*).

### Cytosine Methylation in the Gymnosperms Gi. biloba and Gn. gnemon

**Fig. 6.—:**
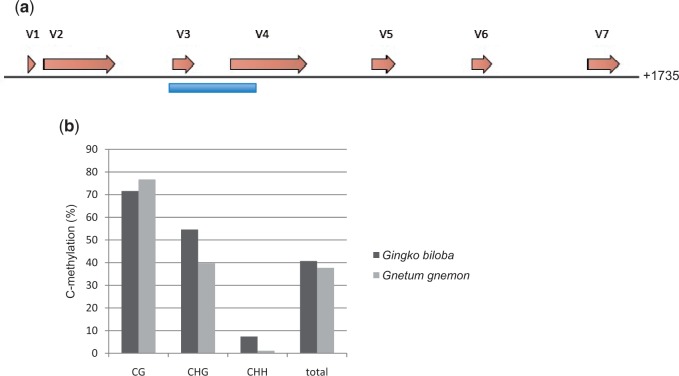
Bisulphite sequencing of part of the 18S rDNA in *Ginkgo biloba* and *Gnetum gnemon* was used to determine the level of C methylation (*a*) Diagrammatic scheme of the *Gn. gnemon* 18 S rDNA unit (Genbank accession number U42416.1) showing the loop regions (V1–V7, brown arrows) and the region selected for bisulphite sequencing (blue line). (*b*) Results of methylation analysis. Note the relatively low level of non-CG methylation in *Gn. gnemon* where only 4/451 CHH sites (0.9%) were methylated.

Since DCL2 was missing in *Gn. gnemon* and *W. mirabilis*, and it is thought to interact with RdDM in the noncanonical methylation of cytosine (involving RDR6; Nuthikattu et al. 2013), we conducted bisulphite sequencing of the 18S rDNA in *Gn. gnemon* and *Gi. biloba* to compare levels of CHH methylation. Figure 6*b* shows that in both species CG and CHG methylation levels were high, but CHH methylation was very low in *Gn. gnemon*.

To further study methylation patterns, we used Southern hybridization and an 18 S rDNA probe against restricted genomic DNA (using methylation-sensitive and insensitive isoschizomers) from the gymnosperms *Gn. gnemon*, *Gi. biloba*, and the angiosperm *Nicotiana tabacum* (chosen as a control because the methylation status of its rDNA has been extensively studied; Lim et al. 2000). We revealed more extensive digestion of *Gn. gnemon* DNA with MspI (sensitive to CHG methylation) compared with the other species (supplementary fig. S4, Supplementary Material online, red circles). This confirmed a relative undermethylation of *Gn. gnemon* rDNA. In contrast, the fraction of rDNA resistant to digestion with methylation-sensitive enzymes (supplementary fig. S4, Supplementary Material online, red bars) was relatively high in *Gi. biloba* and *N. tabacum*, indicating dense methylation of their units.

## Discussion

### Differences in RdDM Pathway Genes across Land Plants

**Fig. 7.—:**
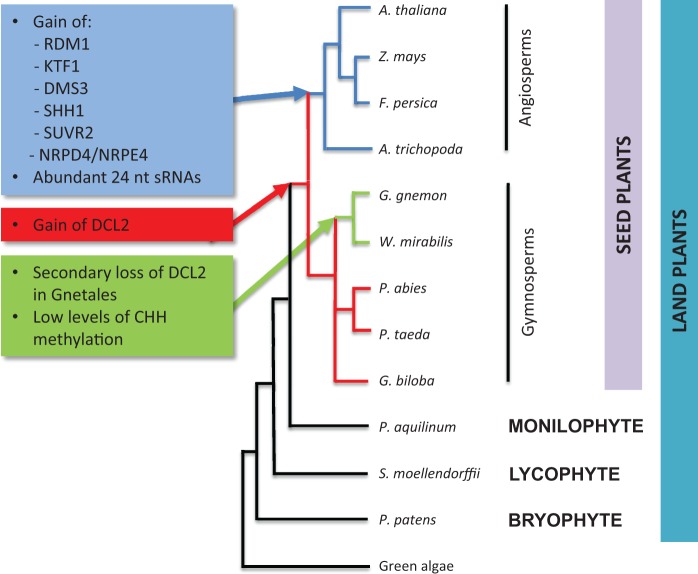
Synthesis of data showing likely origin of gene families associated with the RdDM pathway in the evolution of land plants. The summary tree topology was based on Mathews (2009).

Six proteins were identified in our analyses that were absent or missing outside angiosperms (fig. 7), these are NRPD4/NRPE4, SHH1, RDM1, DMS3, KTF1, and SUVR2. All are involved in phases (1) and (2) of the canonical RdDM pathway (figs. 1 and 2 and supplementary table S1, Supplementary Material online). Collectively, the data suggest that phases (1) and (2) of the RdDM pathway have diverged between the different land plant groups whereas phase (3), which is involved in chromatin remodeling, is the most highly conserved part of the pathway. The other proteins of phases (1) and (2) that we analyzed were found across land plants, perhaps with variant functions outside the seed plants (as in DCL, see below).

NRPD4/NRPE4 is known to function as part of the RNA Pol IV and Pol V complexes. It is encoded by the same gene and is distinct from the NRPB4 subunit of RNA polymerase II (Pol II) in *Ar. thaliana* (He, Hsu, Pontes, et al. 2009; Ream et al. 2009). NRPD4/NRPE4 forms subcomplexes with NRPD7 and NRPE7 in Pol IV and Pol V, respectively (Ream et al. 2009). Pol IV and Pol V are central to the RdDM pathway and probably to its evolution (Matzke et al. 2015). Previously, it was suggested that NRPD4 evolved after the divergence of *Ph. patens* and before angiosperms (Tucker et al. 2010). Our data extend these findings by showing that NRPD4/E4 diverged with the angiosperms.

Pol IV is thought to be recruited to a subset of target loci for siRNA production by the protein SHH1 which recognizes and binds to H3 histones when they are unmethylated at lysine 4 (=H3K4) and methylated at lysine 9 (=H3K9), that is, markers of heterochromatin production (Law et al. 2013; Zhang, Ma, et al. 2013; Matzke et al. 2015). Our failure to detect SHH1 outside angiosperms is consistent with the lack of the NRPD4/NRPE4 subunits of Pol IV.

RDM1 is reported to be needed for Pol V function (Matzke et al. 2015) and is currently understood to interact with the Pol V pathway in phase (2) of the RdDM pathway in two ways: (a) acting as a homodimer protein bridging between AGO4 and DRM2 in the de novo methylation step (Gao et al. 2010; Sasaki et al. 2014), and (b) acting as a monomer in the DDR complex (together with DRD1 and DMS3) that facilitates Pol V transcription (Law et al. 2010). Certainly, Arabidopsis *rdm1* mutants show a nearly complete loss of DNA methylation via the RdDM pathway (Gao et al. 2010; Stroud et al. 2013;Sasaki et al. 2014). Previously Matzke et al. (2015) noted that RDM1 was restricted to angiosperms, and we confirm this in our taxonomically more diverse survey, which includes representatives from all major land plant groups. The phylogenetic tree inferred from RDM1 sequences from 35 angiosperm species illustrates that sequences from three eudicot families cluster into discrete, highly supported, clades (fig. 4).

Overall, it appears that the specialized components of both Pol IV and Pol V pathways may only be present in angiosperms (fig. 7).

The protein SUVR2 was also shown to be restricted to angiosperms (fig. 2 and supplementary table S1, Supplementary Material online). It is a putative histone methyltransferase that is not directly required for the generation of siRNAs by the RdDM pathyway, but was recently shown to be required for DRM2 establishment and for maintaining methylation downstream of siRNA biogenesis (Stroud et al. 2013).

The final angiosperm specific protein is KTF1, a transcription factor that plays a role in phase (2) of the RdDM pathway by coordinating transcriptional elongation with chromatin modifications and pre-mRNA processing via interactions with AGO4 (He, Hsu, Zhu, et al. 2009). It was previously reported to be restricted to angiosperms (Matzke et al. 2015), consistent with findings from the more extensive survey here.

### DCL Proteins

DCL proteins are multidomain endoribonucleases, which “dice” or cut prematured long double stranded RNAs into sRNAs (Bernstein et al. 2001; Liu et al. 2009; Matzke et al. 2015). The number of DCL family members varies among different organisms and patterns of evolution across eukaryotes, including an alga and three angiosperms, have been discussed previously (Margis et al. 2006). In *Ar. thaliana* there are four DCL gene paralogues (DCL1–DCL4) (Schauer et al. 2002), but in other eukaryotic groups the numbers can vary from one to more than four types (Bernstein et al*.* 2001; Liu et al*.* 2009). Our analysis showed that only DCL1 was found in all the land plant lineages examined, which suggests it is the most highly conserved. In *Ar. thaliana*, this protein has a role in generating 21 nt miRNAs involved in posttranscriptional regulation of their target genes.

The isolated absences of other DCL family members in our analysis (e.g., DCL3 in *W. mirabilis* and *Pt. aquilinum* and DCL4 in *Gn. gnemon* and *S. moellendorffii*) may have arisen because the gene transcripts were not sequenced or detected by us. We have therefore put more weight on our findings where there is strong phylogenetic signal in the patterns of gene losses and gains (fig. 3).

In our analysis, DCL2 was only detected in species belonging to the seed plants (fig. 3), although it was not found in the two species of the gymnosperm order Gnetales examined (i.e., *W. mirabilis* and *Gn. gnemon*; fig. 3). It is therefore possible that DCL2 sequences have been secondarily lost with the divergence of these species in Gnetales (fig. 7). DCL2 is thought to be involved in RNA-mediated virus resistance and is associated with the production of 22 nt sRNAs. There may also be interactions between the posttranscriptional gene silencing pathway that targets RNA polymerase II-transcribed genes, including newly transposed retroelements, and the noncanonical methylation of cytosines in the RdDM pathway (Nuthikattu et al. 2013). The latter involves the activities of DCL2 and DCL4 to generate 21 and 22 nt sRNAs. Bisulphite sequencing *of Gn. gnemon* revealed unusually low levels of CHH methylation in 18S rDNA sequences compared with *Gi. biloba*, which does have DCL2 (fig. 6*b* and supplementary fig. S4, Supplementary Material online). Such a result is consistent with an absence of interaction of DCL2 with RdDM in *Gn. gnemon*. If so, the absence of DCL2 outside the seed plants could have similar consequences on the degree of methylation at noncanonical cytosines.

DCL3, which generates 24 nt sRNAs and is directly involved in the canonical RdDM pathway (fig. 1), was found in all plant groups except in the monilophyte studied (fig. 3). Although previous studies failed to detect 24 nt sRNAs in conifers (Dolgosheina et al. 2008), recently they were reported to be present in some tissues of *Cunninghamia lanceolata* (Wan et al. 2012), *Pic. abies* (Nystedt et al. 2013), and *Larix leptolepis* (Zhang, Wu, et al. 2013), consistent with the results presented here. Indeed our survey of the sRNAs generated across land plants shows that all species have a fraction of sRNAs that are 24 nt long although it is only in the angiosperms that these comprise the major fraction of sRNAs (fig. 5). The observation that 24 nt sRNAs were present in the monilophyte *Pt. aquilinum* (fig. 5) may indicate that we have simply failed to find DCL3 in the transcriptome data currently available, rather than the gene being absent from their genomes.

DCL4 is thought to be involved in trans-acting RNA metabolism and post-transcriptional gene regulation, generating 21 nt sRNAs. We found DCL4 in all land plant lineages except the lycophyte studied (table 2 and fig. 3).

In consideration of missing genes in the pathway it must be noted that there is redundancy in function between these DCL families, which results in limited phenotypes in knock-out experiments (Andika et al. 2015). This means that the losses of particular DCL families may be functionally compensated for by the activity of another DCL family member.

### Influence of Different Epigenetic Machinery on Genome Structures

The primary role of RdDM is considered to be the epigenetic silencing of repeats, predominantly retroelements across the genome. This silencing process leads to chromatin remodeling or heterochromatinization, which typically renders the repeats transcriptionally silent (Matzke and Mosher 2014; Matzke et al. 2015). For example, among angiosperms it is known that modifications to, or breakdown of, the RdDM pathway can lead to repeat amplification, as shown, for example, by the inactivity of an orthologue of RDR2 in *Z. mays* resulting in enhanced transposon activity (Woodhouse et al. 2006).

The differences in the epigenetic machinery among representatives of the major land plant groups we show here might potentially influence the evolutionary dynamics of their genomes. Angiosperms are thought to have dynamic genome structures compared with gymnosperms, with a higher level of turnover of retroelements (Leitch and Leitch 2012), at least in those species with a small genome (cf. Kelly et al. 2015). Angiosperms are also remarkable among comparably sized eukaryotic groups in terms of their genome size diversity. Not only do they have the largest range for any comparable group—varying approximately 2,400-fold (1C = 0.063–152.23 pg), but the distribution of genome sizes is skewed towards small genomes, with the modal and median values being just 1C = 0.6 pg and 2.5 pg, respectively (Leitch and Leitch 2013).

To determine if angiosperms with large genomes have anything unusual in their RdDM pathway we analyzed *F. persica*, which has an extraordinary large genome size for any eukaryote (1C = 41.21 pg, Kelly et al. 2015), nearly 300 times that of *Ar. thaliana.* Previously, in a study of a related species (*F. imperialis*; 1C = 43 pg), we identified a pararetrovirus-like repeat sequence (FriEPRV) which was estimated to be present in approximately 21,000 copies, accounting for 0.4% of its genome (Becher et al. 2014). We showed high levels of cytosine methylation and an abundance of 24 nt sRNA reads that mapped exclusively to the repeat, a result which did not suggest anything unusual in the RdDM pathway. Nevertheless, here we failed to detect NRPD1, SUVR2, and SHH1 (supplementary table S1, Supplementary Material online) in *F. persica*. Potentially, KTF1 is also missing since the OrthoMCL group protein isolated lacks GW/WG motifs, which function to interact with AGO4 and siRNAs (He, Hsu, Zhu, et al. 2009). However, for this protein we cannot rule out incomplete assembly (supplementary fig. S3, Supplementary Material online). Similarly, we only found two domains for DCL4 (supplementary table S4, Supplementary Material online), although this too might point towards an incomplete assembly. Nevertheless, collectively, it remains possible that there is divergence in particular components of the RdDM pathway in *Fritillaria*, which perhaps impacts on the amplification and elimination of different types of repeat in the genome. If so, this may contribute to the observation that *Fritillaria* genomes comprise a high diversity of highly heterogeneous repeats, each representing a rather small proportion of the genome (Kelly et al. 2015). Such a pattern of repeats may also be present in other species with large genomes (Metcalfe and Casane 2013). This pattern differs from that generally found in species with small genomes, where amplification of one or a few repeat families can result in the contrasting genome sizes observed (Grover and Wendel 2010; Bennetzen and Wang 2014).

In contrast to angiosperms, gymnosperms have relatively limited genome size variation (just 16-fold overall, 2.25–36.00 pg) despite having the highest proportion of species with recorded DNA C-values (∼25% of species; Leitch and Leitch 2012, 2013). In addition, the mode and median genome size values are significantly higher compared with angiosperms (gymnosperm mode 1C = 10.0 pg, median 1C = 7.9 pg and mean 1C = 18.6 pg). Such differences, coupled with the heterogeneous repeat profiles of the Coniferales species examined (Kovach et al. 2010; Nystedt et al. 2013), could also be related to differences observed in the epigenetic machineries. Potentially in angiosperms RdDM pathways evolved as another, or alternative, layer of transposon proliferation control not found in other land plant groups. In angiosperms, it is thought that activated transposons (transcribing RNA) are resilenced through RdDM. However, we are unaware of evidence for an active transposon in gymnosperms, despite their large genomes, whereas there are many examples in angiosperms (Lisch 2013). Possibly gymnosperms and other land plants have other/alternative mechanisms to silence transposons, such as an elevated frequency of C to T mutation of noncoding, highly methylated repeats.

Available cytological data in monilophytes, lycophytes, and bryophytes point to further differences with seed plants in patterns of genome organization (Leitch and Leitch 2013). Sadly, however, the lack of extensive genomic data for these land plant groups precludes generalizations about their genome dynamics and the role that epigenetics may play. It is clear that more molecular studies are needed to probe the role of RdDM in contributing to the contrasting genomic profiles observed across land plants.

## Supplementary Material

Supplementary data file S1, figures S1–S4, and tables S1–S5 are available at *Genome Biology and Evolution* online (http://www.gbe.oxfordjournals.org/).

Supplementary Data
