# Supplementary material for: Angiosperms Are Unique among Land Plant Lineages in the Occurrence of Key Genes in the RNA-Directed DNA Methylation (RdDM) Pathway
Source: Genome Biol Evol. 2015 Sep 2;7(9):2648–62. doi: 10.1093/gbe/evv171 (PMC4607528; doi:10.1093/gbe/evv171)
Supplement: Supplementary Data [file supp_7_9_2648__index.html]

Angiosperms are Unique Amongst Land Plant Lineages in the Occurrence of Key Genes in the RNA Dependent DNA Methylation (RDDM) Pathway — Angiosperms Are Unique among Land Plant Lineages in the Occurrence of Key Genes in the RNA-Directed DNA Methylation (RdDM) Pathway — Supplementary Data 

# Angiosperms Are Unique among Land Plant Lineages in the Occurrence of Key Genes in the RNA-Directed DNA Methylation (RdDM) Pathway

## Supplementary Data

files

- Supplementary Data - pdf file
- Supplementary Data - docx file
