# Supplementary material for: Angiosperms Are Unique among Land Plant Lineages in the Occurrence of Key Genes in the RNA-Directed DNA Methylation (RdDM) Pathway
Source: Genome Biol Evol. 2015 Sep 2;7(9):2648–62. doi: 10.1093/gbe/evv171 (PMC4607528; doi:10.1093/gbe/evv171)

SUPPLEMENTARY DATA

ANGIOSPERMS ARE UNIQUE AMONGST LAND PLANT LINEAGES IN THE OCCURRENCE OF KEY GENES IN THE RNA DEPENDENT DNA METHYLATION (RdDM) PATHWAY

Lu Ma[1], Andrea Hatlen[1], Laura J. Kelly[1], Hannes Becher[1], Wencai Wang[1], Ales Kovarik[2], Ilia J. Leitch[3] and Andrew R. Leitch[1]

SUPPLEMENTARY TABLES

Table S1. Summary of results from the OrthoMCL analysis to find RdDM pathway genes in 12 representative species of land plants. A summary of the current understanding of phylogenetic relationships between land plants studied is illustrated at the top; angiosperms are in blue. The numbers in the table indicate presence (1) or absence (0), the later meaning a sequence is missing or was not detected. Cells labelled with an asterisk are those proteins subsequently shown to be false positives. The protein families highlighted in blue are those thought to be restricted to angiosperms, either through this OrthoMCL analysis, or from additional data (see results), while protein families of the RdDM pathway found across all land plant groups are highlighted in green.

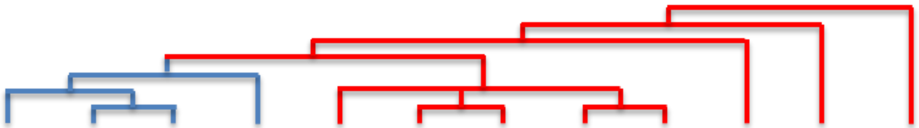

Protein of interest	Reference protein	AGI number of reference protein	*A. thaliana*	*Z. mays*	*F. persica*	*A. trichopoda*	*G. biloba*	*G. gnemon*	*W. mirabilis*	*P. abies*	*P. taeda*	*P. aquilinum*	*S. moellendorffii*	*P. patens*
NUCLEAR RNA POLYMERASE D1(NRPD1)	NRPD1	AT1G63020	1	1	0	1	1	0	0	1	0	0	1	1
NRPE1	NRPE1	AT2G40030	1	1	1	0	1	1	1	1	0	1	0	1
NRPD2/NRPE2	NRPD2/NRPE2	AT3G23780	1	1	1	1	1	1	1	1	1	1	1	1
**NRPD4/NRPE4**	**NRPD4/NRPE4**	AT4G15950	1	1	1	1	0	0	0	0	0	0	0	0
NRPE5	NRPE5	AT3G57080	1	1	1	1	1	1	1	0	0	0	0	0
NRPE9B	NRPE9B	AT4G16265	1	1	1	1	1	1	1	1	1	1	1	1
NRPB1	NRPB1	AT4G35800	1	1	1	1	1	1	1	1	1	1	1	1
RNA-DEPENDENT RNA POLYMERASE (RDR) family	RDR2	AT4G11130	1	1	1	1	1	1	1	1	1	1	1	1
DICER-LIKE (DCL) family	DCL3	AT3G43920	1	1	1	1	1	1	1	1	1	1	1	1
HUA ENHANCER (HEN) family	HEN1	AT4G20910	1	1	1	1	1	1	1	1	1	1	1	1
ARGONAUTE (AGO) family	AGO4	AT2G27040	1	1	1	1	1	1	1	1	1	1	1	1
CLASSY 1 (CLSY1)	CLSY1	AT3G42670	1	1	1	1	1	0	0	1	1	0	0	0
DEFECTIVE IN RNA-DIRECTED DNA METHYLATION 1 (DRD1)	DRD1	AT2G16390	1	1	1	1	1	1	1	1	1	1	0	1
**DEFECTIVE IN MERISTEM SILENCING 3 (DMS3)**	**DMS3**	AT3G49250	1	1	1	1	1*	1*	1*	1*	0	1*	1*	0
**RNA-DIRECTED DNA METHYLATION 1 (RDM1)**	**RDM1**	AT3G22680	1	1	1	1	0	0	0	0	0	0	0	0
**KOW DOMAIN-CONTAINING TRANSCRIPTION FACTOR 1 (KTF1)**	**KTF1**	AT5G04290	1	1	1*	1	0	0	0	0	0	0	0	0
INVOLVED IN *DE NOVO* 2 (IDN2) and its paralogs	IDN2	AT3G48670	1	1	1	1	1	1	1	1	0	1	0	1
DMS4	DMS4	AT2G30280	1	1	1	0	1	1	1	1	1	1	0	1
DOMAINS REARRANGED METHYLTRANSFERASE (DRM) family	DRM2	AT5G14620	1	1	1	1	1	0	0	1	1	1	1	1
SUVH family	SUVH2	AT2G33290	1	1	1	1	1	1	1	1	1	0	0	0
**SUVR family**	**SUVR2**	AT5G43990	1	0	0	0	0	0	0	0	0	0	0	0
MICRORCHIDIA (MORC) family	MORC1	AT4G36290	1	1	1	1	1	1	1	1	0	1	1	1
**SAWADEE HOMEODOMAIN HOMOLOGUE 1 (SHH1)**	**SHH1**	AT1G15215	1	0	0	0	0	0	0	0	0	0	0	0
HISTONE DEACETYLASE (HDA) family	HDA6	AT5G63110	1	1	1	1	1	1	1	1	1	1	1	1
JUMONJI (JMJ) family	JMJ14	AT4G20400	1	1	1	1	1	1	1	1	1	0	1	1
LYSINE-SPECIFIC HISTONE DEMETHYLASE (LDL) family	LDL1	AT1G62830	1	1	1	1	1	1	1	1	1	0	1	1
UBIQUITIN-SPECIFIC PROTEASE (UBP26)	UBP26	AT3G49600	1	1	1	1	1	1	1	1	1	1	1	1
Additional factors														
NEEDED FOR RDR2-INDEPENDENT DNA METHYLATION (NERD)	NERD	AT2G16485	1	1	1	1	1	1	1	1	1	1	0	1
CHROMOMETHYLASE (CMT) family	CMT2	AT4G19020	1	1	1	1	1	0	1	1	1	1	1	1
METHYLTRANSFERASE (MET) family	MET1	AT5G49160	1	1	1	1	1	1	1	1	1	1	1	1
DECREASED DNA METHYLATION 1 (DDM1)	DDM1	AT5G66750	1	1	1	1	1	1	1	1	1	1	1	1

Table S2. DMS3-like proteins identified by OrthoMCL. Only those proteins highlighted in yellow pass the thresholds that were used to distinguish DMS3 orthologues. These protein sequences are in FASTA format in Supplementary Data File 1.

Species	Protein ID	Protein size (amino acids)	5' motif contains Histidine kinase-like ATPase	Protein passes size filter (i.e. > 150 bp and < 700bp)	Transitive Consistence Score
Arabidopsis thaliana	ATHA|AT3G49250.1	422	No	Yes	70
	ATHA|AT5G24280.1	1600	Yes		
Amborella trichopoda	ATRI|evm_27.model.AmTr_v1.0_scaffold00021.223	392	No	Yes	74
	ATRI|evm_27.model.AmTr_v1.0_scaffold00021.222	1487	Yes		
Fritillaria persica	FPER|m.3368	713	Yes		
	FPER|m.8619	720	No		
	FPER|m.15982	393	No	Yes	72
Zea mays	ZMAY|GRMZM2G309152_P02	407	No	Yes	
	ZMAY|GRMZM2G309152_P01	407	No	Yes	72
Ginkgo biloba	GBIL|m.62448	1633	Yes		
	GBIL|m.62451	578	Yes		
	GBIL|m.62450	1065	No		
	GBIL|m.62452	1633	Yes		
	GBIL|m.62454	1633	Yes		
	GBIL|m.62456	1429	No		
	GBIL|m.62458	806	No		
	GBIL|m.62460	1633	Yes		
Gnetum gnemon	GMON|m.25834	261	No	Yes	16
	GMON|m.26467	139	No		
	GMON|m.4488	288	No	Yes	79
	GMON|m.6709	520	No	Yes	67
Welwitschia mirabilis	WMIR|m.12103	174	No	Yes	82
	WMIR|m.10312	1419	Yes		
Picea abies	PABI|MA_10426383g0010	415	No	Yes	16
	PABI|MA_121638g0010	122	No		
	PABI|MA_217357g0010	278	No	Yes	14
	PABI|MA_8123075g0010	122	No		
	PABI|MA_86519g0010	122	No		
Pteridium aquilinum	PAQU|m.27000	235	Yes		
	PAQU|m.26738	374	No	Yes	10
Selaginella moellendorffii	SMOE|432347	1233	Yes		

Table S3. Proteins with similarity to KTF1 identified in our OrthoMCL analysis and in the OrthoMCL Viridiplantae database. The presence of domains characteristic of KTF1 (i.e. KOW and NGN domains) were identified using InterPro (http://www.ebi.ac.uk/interpro/).

Methods	Species	Protein ID	Protein size (amino acids)	WG/GW motif present	Position of NGN domain if present	Position of KOW domain if present	Position of KOW domain if present	Position of KOW domain if present
Our OrthoMCL	*Arabidopsis thaliana*	ATHA|AT5G04290.1	1493	Yes	148 - 231	239 - 266	501 - 528	607 - 634
	Amborella trichopoda	ATRI|evm_27.model.AmTr_v1.0_scaffold00002.356	1704	Yes	158 - 241	249 - 276	516 - 543	627 - 654
	Fritillaria persica	FPER|m.26036	599	No	173 - 256	264 - 291	496 - 523	
	Fritillaria persica	FPER|m.26037	470	No	47 - 127	135 - 162	367 - 394	
	Zea mays	ZMAY|GRMZM2G375222_P01	1564	Yes	159 - 242	472 - 499	568 - 595	
	Zea mays	ZMAY|GRMZM2G370715_P01	1499	Yes	95 - 178	400 - 427	496 - 523	
OrthoMCL Viridiplantae database	*Ricinus communis*	rcom|29898.m000064 | suppressor	1548	Yes	Yes	Yes		
	Oryza sativa Japonica group	osat|NP_001058236 | Os06g0652600	519	No*	No*	No*		
	Physcomitrella patens subsp. *patens*	ppat|fgenesh1_pg.scaffold_3544000001	763	A few	No	No		
	Volvox carteri f. *nagariensis*	vcar|XP_002951135	2169	A few	No	No		
	Volvox carteri f. *nagariensis*	vcar|XP_002952158	900	No	No	No		
	Volvox carteri f. *nagariensis*	vcar|XP_002954205	2301	Yes	No	No		
	Micromonas sp. RCC299	micr|ACO69422	613	No	No	No		

* The database probably has a misidentified orthologue, since rice has KTF1 (He XJ et al. 2009. NRPD4, a protein related to the RPB4 subunit of RNA polymerase II, is a component of RNA polymerases IV and V and is required for RNA-directed DNA methylation. Genes Dev. 23:318–330.)

Table S4. Putative DCL orthologues identified in the OrthoMCL analysis. Protein domains were identified using the Pfam protein database (http://pfam.xfam.org/search).

	Protein ID	DEAD			ResIII			Helicase-C			Dicer_dimer			PAZ			RNaseIII			RNaseIII			dsrm			DND1_DSRM			Selected for phylo-genetic analysis
		start	end	length	start	end	length	start	end	length	start	end	length	start	end	length	start	end	length	start	end	length	start	end	length	start	end	length	
1	ATHA|AT1G01040.1				248	415	167	648	765	117	840	928	88	1200	1338	138	1375	1518	143	1594	1707	113	1734	1794	60	1832	1904	72	no
2	ATHA|AT1G01040.2				248	415	167	648	765	117	840	928	88	1201	1339	138	1376	1519	143	1595	1708	113	1735	1795	60	1833	1905	72	yes
3	ATHA|AT3G03300.1	26	191	165				384	490	106	559	638	79	818	958	140	995	1113	118	1185	1296	111							yes
4	ATHA|AT3G03300.2							370	476	106	545	624	79	804	944	140	981	1099	118	1171	1282	111							no
5	ATHA|AT3G03300.3	26	191	165				384	490	106	559	638	79	818	958	140	995	1113	118	1185	1296	111							no
6	ATHA|AT3G43920.1							345	458	113				796	932	136	973	1108	135	1182	1290	108							no
7	ATHA|AT3G43920.2	55	188	133				394	507	113				845	981	136	1022	1157	135	1231	1339	108							yes
8	ATHA|AT3G43920.3	55	203	148				384	497	113				835	971	136	1012	1147	135	1221	1329	108							no
9	ATHA|AT5G20320.1	127	287	160				474	590	116	656	742	86	954	1076	122	1119	1251	132	1327	1436	109				1621	1696	75	yes
10	ATHA|AT5G20320.2				130	275	145	460	576	116	642	728	86	940	1062	122	1105	1237	132	1313	1422	109				1607	1682	75	no
11	ATRI|evm_27.model.AmTr_v1.0_scaffold00016.358				42	202	160	391	502	111	572	652	80	853	979	126	1022	1140	118	1213	1325	112							yes
12	ATRI|evm_27.model.AmTr_v1.0_scaffold00018.3				379	551	172	787	904	117	979	1067	88	1344	1479	135	1516	1664	148	1741	1854	113	1881	1941	60	1965	2038	73	yes
13	ATRI|evm_27.model.AmTr_v1.0_scaffold00026.16							113	228	115	294	377	83	598	709	111	752	884	132	960	1069	109	1096	1159	63	1282	1356	74	yes
14	ATRI|evm_27.model.AmTr_v1.0_scaffold00033.66				33	199	166	399	503	104	576	659	83	900	1031	131	1072	1204	132	1284	1396	112							yes
15	FPER|m.19844							67	184	117	251	322	71																yes
16	FPER|m.31313							161	279	118	349	428	79	615	744	129	787	905	118										yes
17	FPER|m.50359	63	225	162				460	577	117	652	740	88	1020	1156	136	1193	1340	147	1415	1528	113	1555	1615	60	1647	1719	72	yes
18	FPER|m.50361	63	225	162				460	577	117	652	740	88	1020	1156	136	1193	1340	147	1415	1528	113	1555	1615	60	1643	1715	72	no
19	FPER|m.53890													199	331	132	373	505	132	583	692	109							no
20	FPER|m.53892										38	121	83	351	483	132	525	657	132	735	849	114							no
21	FPER|m.53894													199	331	132	373	505	132	583	697	114							no
22	FPER|m.53895													199	331	132				583	692	109							no
23	FPER|m.53900										38	121	83	351	483	132	525	657	132	735	844	109							no
24	FPER|m.53902													199	331	132	373	505	132	583	692	109							no
25	FPER|m.53904										38	121	83	351	483	132	525	657	132	735	844	109							no
26	FPER|m.53906										38	121	83	351	483	132	525	657	132	735	844	109							yes
27	GBIL|m.34136																70	183	113				210	267	57	299	373	74	no
28	GBIL|m.34137				496	663	167	899	1016	117	1091	1179	88	1461	1591	130	1628	1780	152	1856	1969	113				2085	2159	74	yes
29	GBIL|m.34140				496	663	167	899	1016	117	1091	1179	88	1461	1591	130	1628	1780	152	1856	1969	113				2085	2159	74	no
30	GBIL|m.34143				496	663	167	899	1016	117	1091	1179	88	1461	1591	130	1628	1780	152	1856	1969	113				2085	2159	74	no
31	GBIL|m.3525													134	256	122	299	432	133	508	620	112	647	708	61				no
32	GBIL|m.3526				55	210	155	404	522	118	590	673	83																no
33	GBIL|m.3527													134	256	122	299	432	133	508	620	112	647	708	61	878	950	72	yes
34	GBIL|m.3528				55	210	155	404	522	118	590	673	83																no
35	GBIL|m.62494	83	244	161				438	555	117	624	710	86	949	1080	131	1121	1192	71										yes
36	GBIL|m.72744													109	232	123	275	389	114	466	580	114	607	670	63				yes
37	GMON|m.15817																			79	192	113	219	276	57	308	379	71	yes
38	GMON|m.15819																			79	192	113	219	276	57				no
39	GMON|m.17495				88	245	157	482	599	117	674	762	88	1044	1174	130	1211	1357	146										no
40	GMON|m.17497				155	312	157	549	666	117	741	829	88	1111	1241	130	1278	1424	146										yes
41	GMON|m.4118																57	186	129	261	323	62							yes
42	PABI|MA_10429839g0010										10	97	87	322	453	131	495	548	53										yes
43	PABI|MA_10432726g0010																						163	226	63				yes
44	PABI|MA_10436812g0020																									54	128	74	yes
45	PABI|MA_10437243g0010							7	48	41	166	249	83																no
46	PABI|MA_10437243g0020													253	383	130	421	571	150	647	686	39							yes
47	PABI|MA_8664686g0010				459	628	169	864	981	117	1056	1129	73																yes

Table S4 (continued)

	Protein ID	DEAD			ResIII			Helicase-C			Dicer_dimer			PAZ			RNaseIII			RNaseIII			dsrm			DND1_DSRM			Selected for phylo-genetic analysis
		start	end	length	start	end	length	start	end	length	start	end	length	start	end	length	start	end	length	start	end	length	start	end	length	start	end	length	
48	PAQU|m.18096													46	169	123	212	339	127										yes
49	PAQU|m.18097													46	169	123	212	341	129										no
50	PAQU|m.18098													46	169	123	212	328	116										no
51	PAQU|m.20279							138	254	116																			yes
52	PAQU|m.25857													67	198	131	235	390	155										yes
53	PPAT|pacid=28238218				3	164	161	365	473	108	544	627	83	875	1048	173	1086	1233	147	1315	1426	111							yes
54	PPAT|pacid=28238219				3	164	161	365	473	108	544	627	83	875	1048	173	1086	1233	147	1315	1426	111							no
55	PPAT|pacid=28238220							267	375	108	446	529	83	777	950	173	998	1145	147	1227	1338	111							no
56	PPAT|pacid=28246681				31	197	166	385	503	118	574	655	81	862	990	128	1031	1159	128	1235	1345	110							yes
57	PPAT|pacid=28246682				31	197	166	385	503	118	574	655	81	862	990	128	1031	1159	128	1235	1345	110							no
58	PPAT|pacid=28246683							193	311	118	382	463	81	670	798	128	839	967	128	1043	1153	110				1501	1577	76	no
59	PPAT|pacid=28246684										64	145	81	352	480	128	521	649	128	725	835	110				1183	1259	76	no
60	PPAT|pacid=28258649							348	389	41	464	530	66				833	975	142							1178	1250	72	yes
61	PPAT|pacid=28267563				136	295	159	533	650	117	725	813	88	1097	1229	132	1266	1421	155	1497	1608	111	1637	1694	57	1728	1800	72	yes
62	PPAT|pacid=28267564				136	295	159	533	650	117	725	813	88	1097	1229	132	1266	1421	155	1497	1608	111	1637	1694	57				no
63	PTAE|PITA_000010957-RA																243	355	112				382	447	65				yes
64	PTAE|PITA_000010958-RA										98	162	64																yes
65	PTAE|PITA_000017463-RA										67	154	87	395	533	138	575	712	137	791	903	112							yes
66	PTAE|PITA_000024846-RA																16	149	133	228	329	101							yes
67	PTAE|PITA_000036404-RA																2	114	112	190	302	112							yes
68	PTAE|PITA_000041521-RA																11	129	118	205	313	108	341	406	65				yes
69	PTAE|PITA_000050146-RA				459	627	168	863	980	117	1055	1143	88	1424	1554	130	1591	1742	151	1801	1872	71				1919	1993	74	yes
70	SMOE|429802													171	285	114	325	471	146										yes
71	SMOE|444049	358	520	162				705	818	113	888	961	73	1172	1287	115	1326	1450	124	1526	1633	107							no
72	SMOE|448444				367	522	155	706	819	113				1141	1256	115	1295	1419	124	1495	1602	107							yes
73	SMOE|86110				105	259	154	481	598	117	673	761	88	1022	1153	131	1190	1343	153	1419	1532	113	1559	1619	60	1644	1717	73	yes
74	WMIR|m.12712				181	350	169	587	704	117	779	867	88	1153	1283	130	1320	1472	152	1548	1661	113	1688	1745	57	1778	1851	73	yes
75	WMIR|m.18618																88	217	129	291	396	105							yes
76	WMIR|m.21359																42	126	84										yes
77	ZMAY|GRMZM2G001631_P01																65	182	117	255	363	108	390	455	65				yes
78	ZMAY|GRMZM2G024466_P01							2	102	100	168	250	82																yes
79	ZMAY|GRMZM2G040762_P01							52	167	115	242	330	88	592	741	149	778	925	147	1000	1113	113	1140	1198	58	1223	1296	73	yes
80	ZMAY|GRMZM2G301405_P01													142	273	131	314	418	104	490	600	110	628	691	63				yes
81	ZMAY|GRMZM2G413853_P01	36	170	134				336	455	119	525	608	83	838	970	132	1008	1142	134	1222	1332	110							yes
82	ZMAY|GRMZM2G413853_P02	36	170	134				336	455	119	525	608	83	838	970	132	1008	1142	134	1222	1332	110							no
83	ZMAY|GRMZM2G413853_P03							18	137	119	207	290	83	520	652	132	690	824	134	904	1014	110							no
84	ZMAY|GRMZM5G814985_P01																13	147	134	224	335	111							yes
**Additional rice DCLs**																													
	OSAT|LOC_Os01g681201							349	462	113	533	613	80	835	973	138	1012	1143	131	1223	1334	111							yes
	OSAT|LOC_Os04g430501							328	446	118	512	594	82	772	900	128	943	1073	130	1149	1258	109	1285	1349	64				yes
	OSAT|LOC_Os03g029701							627	742	115	817	905	88	1166	1316	150	1353	1498	145	1573	1686	113				1798	1870	72	yes
	OSAT|LOC_Os03g387401	37	204	167				392	499	107	569	648	79	831	965	134	1006	1124	118	1197	1307	110							yes
	OSAT|LOC_Os09g146101	23	111	88										96	229	133	270	388	118	461	568	107	596	658	62				yes
	OSAT|LOC_Os10g344301	327	448	121				327	448	121	516	599	83	829	961	132	999	1133	134	1211	1322	111							yes

Table S5. RDM1-like sequences extracted from the NCBI Protein Reference Sequence database (arranged alphabetically by genus).

Genbank accession number	Species	Family (major taxonomic group)	Abbreviation used in Fig. 4
XP_006839001.1	*Amborella trichopoda*	Amborellaceae (Early-diverging angiosperm)	*A.trich*
XP_002883375.1	*Arabidopsis lyrata* subsp. *lyrata*	Brassicaceae (Eudicots)	*A.lyrat*
NP_188907.2	*Arabidopsis thaliana*	Brassicaceae (Eudicots)	*A.thali*
XP_010227483.1	*Brachypodium distachyon*	Poaceae (Monocots)	*B.dista*
XP_009145304.1	*Brassica rapa*	Brassicaceae (Eudicots)	*B.rapa*
XP_010488301.1	*Camelina sativa*	Brassicaceae (Eudicots)	*C.sativ*
XP_010511601.1	"	"	"
XP_010466557.1	"	"	"
XP_006298700.1	*Capsella rubella*	Brassicaceae (Eudicots)	*C.rubel*
XP_004511378.1	*Cicer arietinum*	Fabaceae (Eudicots)	*C.ariet*
XP_004511377.1	"	"	"
XP_004511382.1	"	"	"
XP_004511381.1	"	"	"
XP_004511455.1	"	"	"
XP_006419996.1	*Citrus clementine*	Rutaceae (Eudicots)	*C.cleme*
XP_006419997.1	"	"	"
XP_006489435.1	*Citrus sinensis*	Rutaceae (Eudicots)	*C.sinen*
XP_008438640.1	*Cucumis melo*	Cucurbitaceae (Eudicots)	*C.melo*
XP_004134127.1	*Cucumis sativus*	Cucurbitaceae (Eudicots)	*C.sativ1*
XP_010024237.1	*Eucalyptus grandis*	Myrtaceae (Eudicots)	*E.grand*
XP_006406137.1	*Eutrema salsugineum*	Brassicaceae (Eudicots)	*E.salsu*
XP_006410306.1	"	"	"
XP_006396697.1	"	"	"
XP_004298016.1	*Fragaria vesca* subsp. *vesca*	Rosaceae (Eudicots)	*F.vesca*
XP_004294547.1	"	"	"
NP_001237231.1	*Glycine max*	Fabaceae (Eudicots)	*G.max*
XP_006590409.1	"	"	"
XP_006590408.1	"	"	"
XP_003610752.1,	*Medicago truncatula*	Fabaceae (Eudicots)	*M.trunc*
XP_003610751.1	"	"	"
XP_010092414.1	*Morus notabilis*	Moraceae (Eudicots)	*M.notab*
XP_009388605.1	*Musa acuminata* subsp. *malaccensis*	Musaceae (Monocots)	*M.malac*
XP_010256138.1	*Nelumbo nucifera*	Nelumbonaceae (Eudicots)	*N.nucif*
XP_009765154.1	*Nicotiana sylvestris*	Solanaceae (Eudicots)	*N.sylve*
XP_009802508.1	"	"	"
XP_009802505.1	"	"	"
XP_009802507.1	"	"	"
XP_009802504.1	"	"	"
XP_009587840.1	*Nicotiana tomentosiformis*	Solanaceae (Eudicots)	*N.tomen*
XP_009587841.1	"	"	"
XP_009587843.1	"	"	"
XP_009587839.1	"	"	"
XP_009602621.1	"	"	"
XP_009602618.1	"	"	"
XP_009602620.1	"	"	"
XP_009602616.1	"	"	"
XP_007157108.1	*Phaseolus vulgaris*	Fabaceae (Eudicots)	*P.vulga*
XP_008804974.1	*Phoenix dactylifera*	Arecaceae (Monocots)	*P.dacty*
XP_002311634.1	*Populus trichocarpa*	Salicaceae (Eudicots)	*P.trich*
XP_002311260.1	"	"	"
XP_008224276.1	*Prunus mume*	Rosaceae (Eudicots)	*P.mume*
XP_008233672.1	"	"	"
XP_007223423.1	*Prunus persica*	Rosaceae (Eudicots)	*P.persi*
XP_009371962.1	*Pyrus* x *bretschneideri*	Rosaceae (Eudicots)	*P.brets*
XP_009369876.1	"	"	"
XP_002517093.1	*Ricinus communis*	Euphorbiaceae (Eudicots)	*R.commu*
XP_004965822.1	*Setaria italica*	Poaceae (Monocots)	*S.itali*
XP_004248831.1	*Solanum lycopersicum*	Solanaceae (Eudicots)	*S.lycop*
XP_004248830.1	"	"	"
XP_004247674.2	"	"	"
XP_006339951.1,	*Solanum tuberosum*	Solanaceae (Eudicots)	*S.tuber*
XP_006360751.1	"	"	"
XP_007034826.1	*Theobroma cacao*	Malvaceae (Eudicots)	*T.cacao*
XP_007034827.1	"	"	"
XP_002279112.2	*Vitis vinifera*	Vitaceae (Eudicots)	*V.vinif*
XP_002278910.1	"	"	"
NP_001170520.1	*Zea mays*	Poaceae (Monocots)	*Z.mays*

SUPPLEMENTARY DATA FILE

Supplementary Data File 1. FASTA format of proteins used in analyses are available at https://goo.gl/PrNKfB.

SUPPLEMENTARY FIGURES

Figure S1. Flowchart illustrating the analytical approach taken.

Figure S2. Unrooted phylogenetic tree depicting relationships between seven putative DMS3 OrthoMCL group proteins in seed plants and the SMC-related protein GMI1 from *Arabidopsis thaliana*. The full protein IDs are given in Table S2 and sequences in FASTA format are provided in Supplementary Data File 1.

Figure S3. KTF1 protein domains in angiosperms identified using InterPro (http://www.ebi.ac.uk/interpro/).

Figure S4. DNA methylation levels were analysed in the 18S rRNA genes of (a) *Ginkgo biloba*, (b) *Gnetum gnemon* and (c) *Nicotiana tabacum* using the methylation-sensitive restriction enzymes *Msp*I (M), *Hpa*II (H) and *Scr*FI (S) and hybridization of restricted DNAs with the 18S rDNA probe (see Figure 6a). The *Msp*I/*Hpa*II isoschizomeres cut at CCGG and are sensitive to CCG and CG methylation, respectively. *Bst*NI (B) and *Scr*FI are nearly isoschizomeric pairs cutting at CCWGG and CCNGG, respectively. *Bst*NI is methylation-insensitive, *Scr*FI is sensitive to methylation of the inner C (CHG methylation). There are more than five target restriction sites in each 18S gene. The probe hybridised to high-molecular-weight bands (red vertical bars) produced by digestion with methylation-sensitive *Hpa*II. This indicated that rDNA sequences are heavily methylated at CG sites in all species. *Msp*I digestion yielded high-molecular-weight fragments in *G. biloba* and *N. tabacum* while in *G. gnemon* the probe hybridised mostly to low-molecular weight bands (circled in red). In all species, the probe hybridised to low-molecular-weight *Bst*NI fragments.

Fig. S1

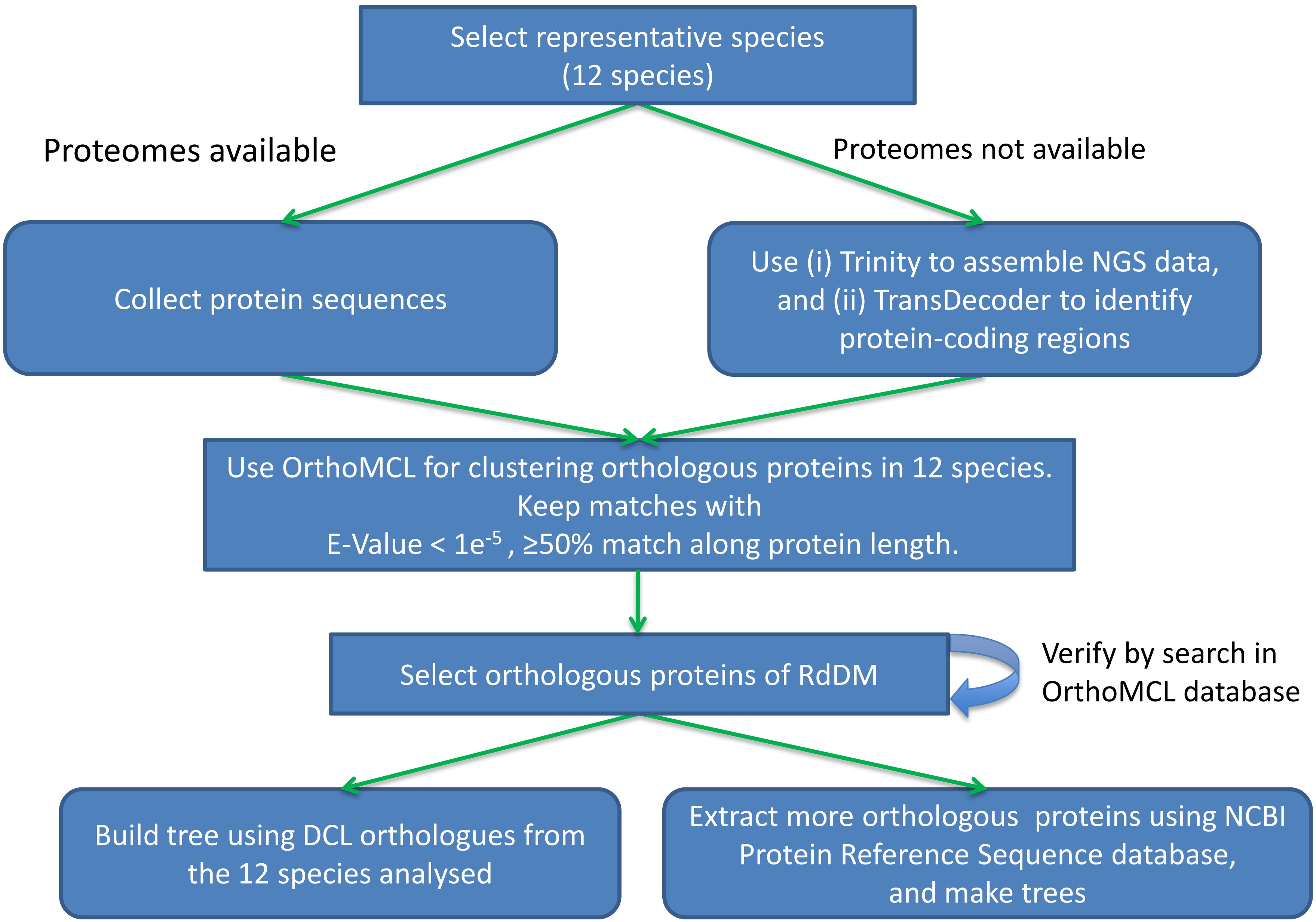

Select representative species
(12 species)
Proteomes available
Proteomes not available
Collect protein sequences
Use (i) Trinity to assemble NGS data, and (ii) TransDecoder to identify protein-coding regions
Use OrthoMCL for clustering orthologous proteins in 12 species.
Keep matches with
E-Value < 1e-5 , ≥50% match along protein length.
Select orthologous proteins of RdDM
Verify by search in OrthoMCL database
Build tree using DCL orthologues from the 12 species analysed
Extract more orthologous proteins using NCBI Protein Reference Sequence database, and make trees

Fig. S2

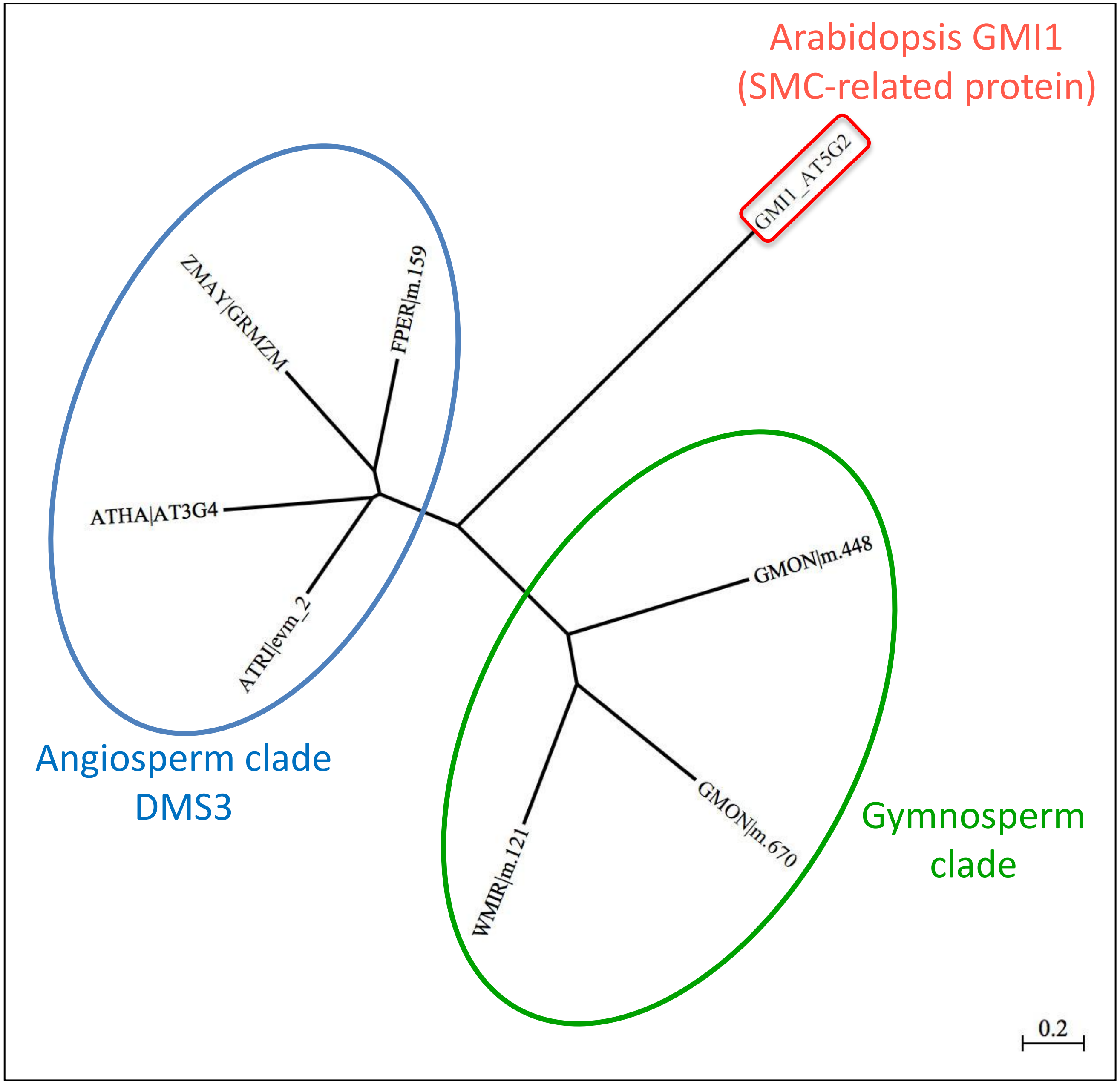
Arabidopsis GMI1
(SMC-related protein)
GMI1_AT5G2
ZMAY|GRMZM
FPER|m.159
ATHA|AT3G4
ATRI|evm_2
GMON|m.448
GMON|m.670
WMIR|m.121
Angiosperm clade
DMS3
Gymnosperm
clade
0.2

Fig. S3

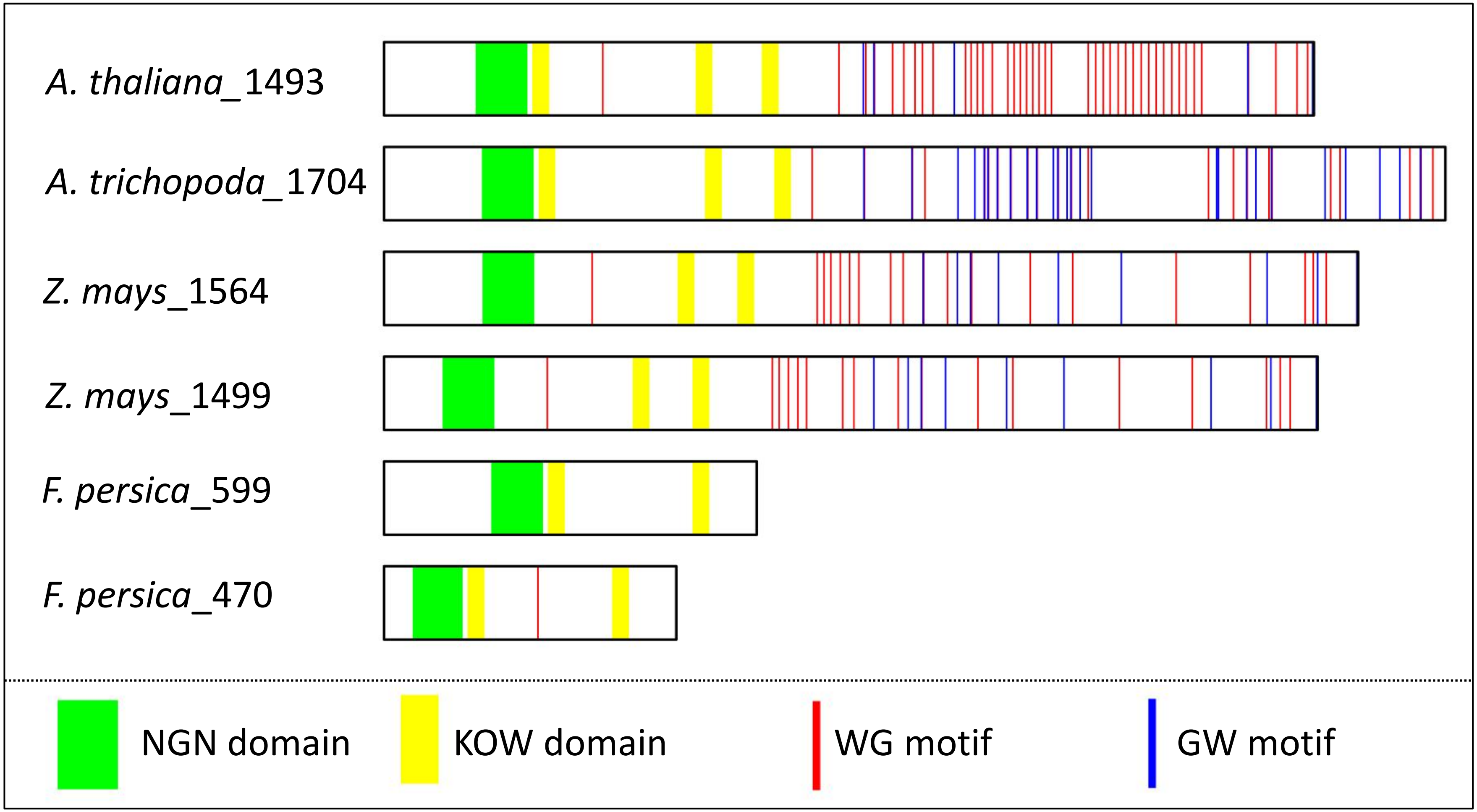

A. thaliana_1493
A. trichopoda_1704
Z. mays_1564
Z. mays_1499
F. persica_599
F. persica_470
NGN domain
KOW domain
WG motif
GW motif

Figure S4

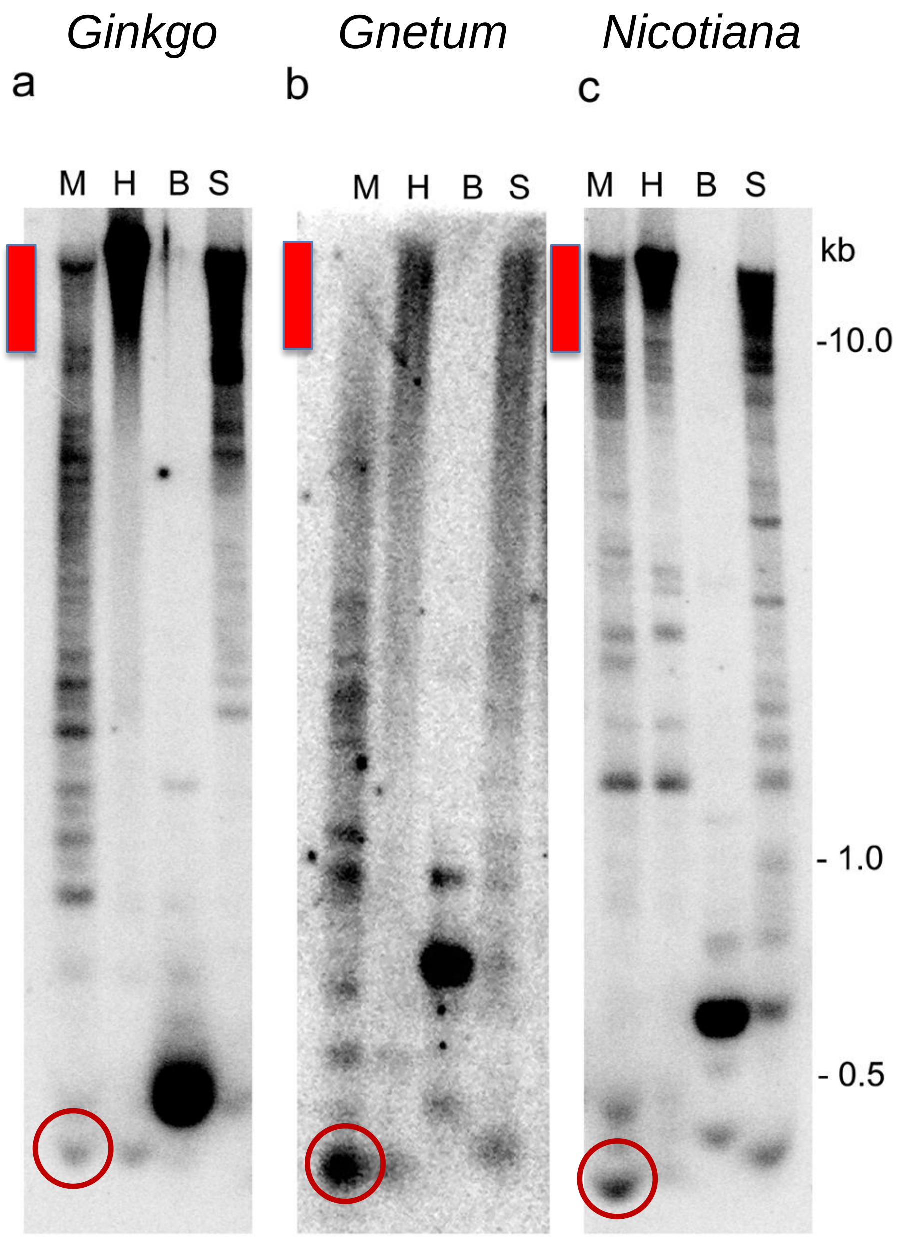

Supplement: Supplementary Data [file supp_evv171_Ma_et_al_Revised_suppl_data_21_Aug_2015.pdf]
