# Supplementary material for: Angiosperms Are Unique among Land Plant Lineages in the Occurrence of Key Genes in the RNA-Directed DNA Methylation (RdDM) Pathway
Source: Genome Biol Evol. 2015 Sep 2;7(9):2648–62. doi: 10.1093/gbe/evv171 (PMC4607528; doi:10.1093/gbe/evv171)
Supplement: Supplementary Data [file supp_evv171_New_Microsoft_Office_Word_Document.docx]

**SUPPLEMENTARY DATA**

**SUPPLEMENTARY FIGURES**

**Figure S1.** Flowchart illustrating the analytical approach taken.

**Figure S2.**  Unrooted phylogenetic tree depicting relationships between seven putative DMS3 OrthoMCL group proteins in seed plants and the SMC-related protein GMI1 from *Arabidopsis thaliana*. The full protein IDs are given in Table S2 and sequences in FASTA format are provided in Supplementary Data File 1.

**Figure S3.** KTF1 protein domains in angiosperms identified using InterPro (<http://www.ebi.ac.uk/interpro/>).

**Figure S4**. DNA methylation levels were analysed in the 18S rRNA genes of (a) *Ginkgo biloba*, (b) *Gnetum gnemon* and (c) *Nicotiana tabacum* using the methylation-sensitive restriction enzymes *Msp*I (M), *Hpa*II (H) and *Scr*FI (S) and hybridization of restricted DNAs with the 18S rDNA probe (see Figure 6a). The *Msp*I/*Hpa*II isoschizomeres cut at CCGG and are sensitive to CCG and CG methylation, respectively. *Bst*NI (B) and *Scr*FI are nearly isoschizomeric pairs cutting at CCWGG and CCNGG, respectively. *Bst*NI is methylation-insensitive, *Scr*FI is sensitive to methylation of the inner C (CHG methylation). There are more than five target restriction sites in each 18S gene. The probe hybridised to high-molecular-weight bands (red vertical bars) produced by digestion with methylation-sensitive *Hpa*II. This indicated that rDNA sequences are heavily methylated at CG sites in all species. *Msp*I digestion yielded high-molecular-weight fragments in *G. biloba* and *N. tabacum* while in *G. gnemon* the probe hybridised mostly to low-molecular weight bands (circled in red). In all species, the probe hybridised to low-molecular-weight *Bst*NI fragments.

**SUPPLEMENTARY TABLES**

**Table S1.** Summary of results from the OrthoMCL analysis to find RdDM pathway genes in 12 representative species of land plants. A summary of the current understanding of phylogenetic relationships between land plants studied is illustrated at the top; angiosperms are in blue. The numbers in the table indicate presence (1) or absence (0), the later meaning a sequence is missing or was not detected. Cells labelled with an asterisk are those proteins subsequently shown to be false positives. The protein families highlighted in blue are those thought to be restricted to angiosperms, either through this OrthoMCL analysis, or from additional data (see results), while protein families of the RdDM pathway found across all land plant groups are highlighted in green.


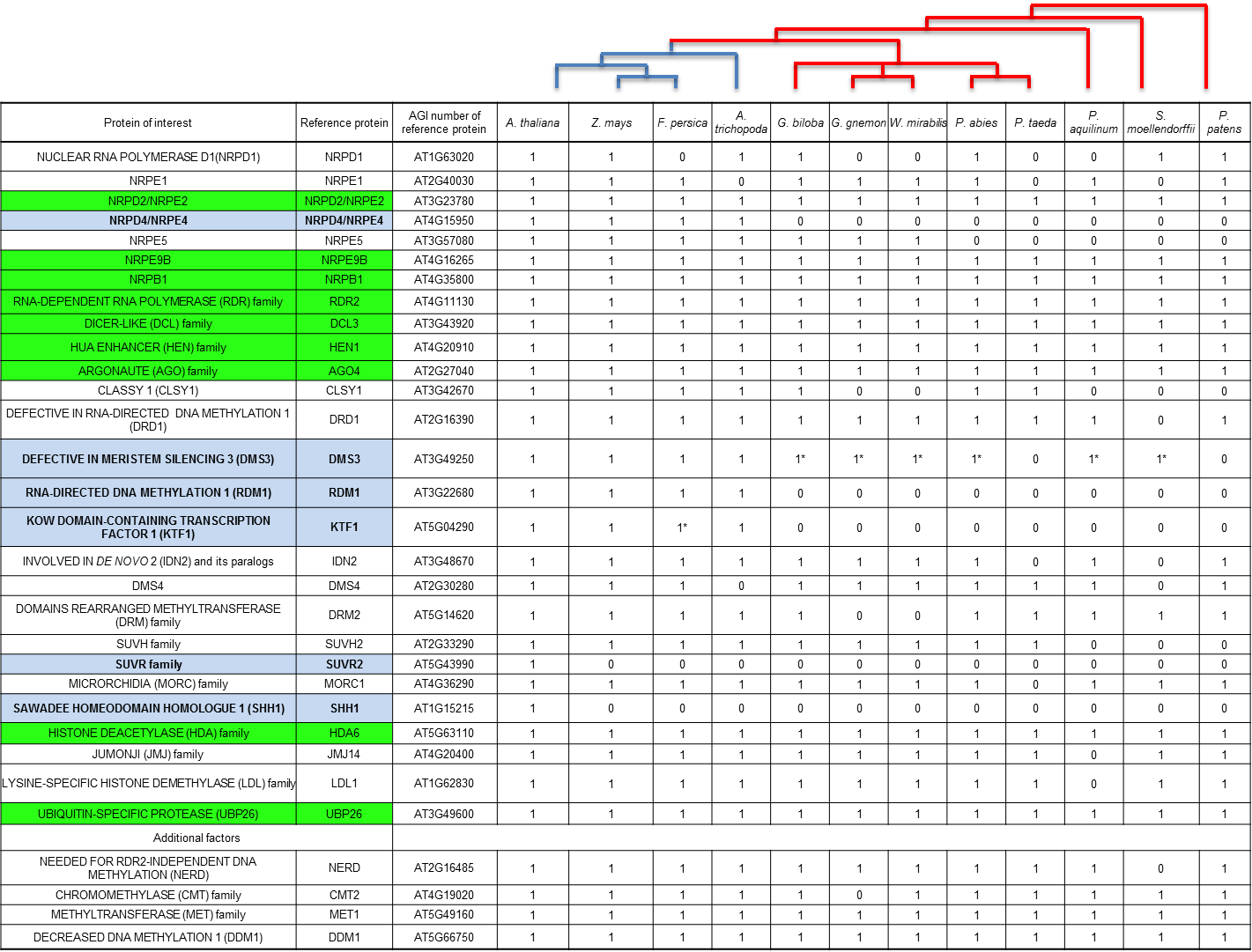


**Table S2.** DMS3-like proteins identified by OrthoMCL. Only those proteins highlighted in yellow pass the thresholds that were used to distinguish DMS3 orthologues. These protein sequences are in FASTA format in Supplementary Data File 1.

**Table S3.** Proteins with similarity to KTF1 identified in our OrthoMCL analysis and in the OrthoMCL Viridiplantae database. The presence of domains characteristic of KTF1 (i.e. KOW and NGN domains) were identified using InterPro (<http://www.ebi.ac.uk/interpro/>).

* The database probably has a misidentified orthologue, since rice has KTF1 (He XJ et al. 2009. NRPD4, a protein related to the RPB4 subunit of RNA polymerase II, is a component of RNA polymerases IV and V and is required for RNA-directed DNA methylation. Genes Dev. 23:318–330.)

**Table S4**. Putative DCL orthologues identified in the OrthoMCL analysis. Protein domains were identified using the Pfam protein database (<http://pfam.xfam.org/search>).

**Table S4 (continued)**

**Table S5.** RDM1-like sequences extracted from the NCBI Protein Reference Sequence database (arranged alphabetically by genus).

**SUPPLEMENTARY DATA FILE**

**Supplementary Data File 1**. FASTA format of proteins used in analyses are available at https://goo.gl/PrNKfB.
